# Manipulating the Hydrogen‐Associated Insulator‐Metal Transition Through Artificial Microstructure Engineering

**DOI:** 10.1002/advs.202510771

**Published:** 2025-10-13

**Authors:** Xuanchi Zhou, Xiaohui Yao, Wentian Lu, Jinjian Guo, Jiahui Ji, Lili Lang, Guowei Zhou, Chunwei Yao, Xiaomei Qiao, Huihui Ji, Zhe Yuan, Xiaohong Xu

**Affiliations:** ^1^ Key Laboratory of Magnetic Molecules and Magnetic Information Materials of Ministry of Education & School of Chemistry and Materials Science Shanxi Normal University Taiyuan 030031 China; ^2^ Research Institute of Materials Science Shanxi Key Laboratory of Advanced Magnetic Materials and Devices Shanxi Normal University Taiyuan 030031 China; ^3^ National Key Laboratory of Materials for Integrated Circuits Shanghai Institute of Microsystem and Information Technology Chinese Academy of Sciences Shanghai 200050 China; ^4^ Interdisciplinary Center for Theoretical Physics and Information Sciences Institute of Nanoelectronic Devices and Quantum Computing Fudan University Shanghai 200433 China

**Keywords:** correlated oxides, electronic phase transition, hydrogen diffusion, ionic evolution, microstructure engineering

## Abstract

Hydrogen‐associated filling‐controlled Mottronics within electron‐correlated system provides a groundbreaking paradigm to explore exotic physical functionality and phenomena. Dynamically controlling hydrogen‐related phase transitions through external fields offers a promising route for designing protonic devices in multidisciplinary fields but faces high‐speed bottlenecks owing to slow bulk diffusion of hydrogens. Here, a promising pathway is presented to kinetically expedite the electronic state evolution in VO_2_ system by taking advantage of artificial microstructure design. Typically, inclined domain boundary configuration and *c*_R_‐faceted preferential orientation, simultaneously realized in VO_2_/Al_2_O_3_ (1$\bar{1}$02) heterostructure, significantly lower the diffusion barrier through creating an unobstructed conduit for hydrogen diffusion. As a result, the achievable switching speed through hydrogenation outperforms that of counterpart grown on widely‐utilized *c*‐plane Al_2_O_3_ substrate by 2–3 times, with resistive switching concurrently improved by an order of magnitude. Of particular interest, an anomalous uphill hydrogen diffusion observed for VO_2_ with a diffusion highway fundamentally deviates from basic Fick's law, unveiling a deterministic role of hydrogen spatial distribution in tailoring electronic state evolution. The present work not only provides a powerful tuning knob for manipulating ionic evolution, endowing with great potential in designing advanced protonic devices, but also deepens the understanding of hydrogen‐associated insulator‐metal transition in electron‐correlated systems.

## Graphical abstract

Establishing an unobstructed conduit for hydrogen diffusion via the artificial design of material microstructure associated with boundary configuration and crystal orientation offers a powerful tuning knob for extensively accelerating the proton mobility and enhancing electronic phase modulations, thereby advancing the high‐speed protonic device applications.

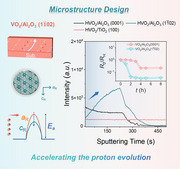

## 1 Introduction

Ionic control of correlated oxides opens a pioneering paradigm to dynamically manipulate multiple physical functionalities and discover exotic physical phenomena, enabling multidisciplinary applications in energy conversions, artificial intelligence, superconductivity, bio‐sensing, and correlated electronics.^[^ ^1^, ^2^, ^3^, ^4^, ^5^, ^6^, ^7^, ^8^ ^]^ Among the existing ionic species, hydrogen ions or protons (e.g., H^+^), characterized by the smallest ionic radius and ultrahigh mobility, can be exploited to drive topotactic phase modulations within correlated oxide system in a more reversible and controllable fashion.^[^ ^9^ ^]^ Incorporating hydrogens into the lattice of correlated oxides can bring in an additional ion degree of freedom to promote the complex interplay of charge, lattice, spin, and orbital degrees of freedom, serving as a promising platform to probe ion‐electron‐lattice coupling.^[^ ^4^, ^10^, ^11^, ^12^, ^13^ ^]^ As a representative case, hydrogenation provides a powerful pathway to drive Mott phase modulations in an extensive collection of *d*‐orbital correlated oxide system, for example, VO_2_, SrRuO_3,_ and *Re*NiO_3_, leading to a rich spectrum of magnetoelectric states.^[^ ^14^, ^15^, ^16^, ^17^, ^18^, ^19^, ^20^, ^21^, ^22^, ^23^ ^]^ The protonic control over the electronic states of correlated oxides is closely associated with the hydrogen‐associated band‐filling process, during which doped electron carriers adjust the band structure to upset the stability of the correlated electronic ground state.^[^ ^24^ ^]^ More importantly, manipulating the hydrogen‐mediated phase transitions using external fields provides a fertile ground to develop proton‐based device applications.^[^ ^25^, ^26^ ^]^ An archetypical example is the protonic transistor device, in which the voltage‐actuated proton evolution can reversibly adjust the channel conductance, triggering multi‐state resistive switching.^[^ ^27^, ^28^ ^]^

Nevertheless, the relatively slow hydrogenation kinetics resulting from the bulk diffusion of hydrogens poses a significant challenge to design high‐speed electronic devices using proton evolution. Conventionally, the hydrogen diffusion within the lattice of correlated oxides is driven by the hydrogen concentration gradient, following Fick's laws of diffusion, the kinetics of which is progressively depressed with the diffusion length away from the hydrogen source.^[^ ^9^ ^]^ One feasible pathway for promoting proton evolution is to artificially engineer the material microstructure that establishes a highway for hydrogen diffusion to lower the diffusion barrier. Typically, intercalated hydrogens, acting as interstitial point defects, readily interact with extended planer defects in correlated oxides, and therefore an inclined or vertical boundary configuration accelerates the hydrogen diffusion, in comparison with the horizontally aligned one.^[^ ^24^, ^29^, ^30^, ^31^, ^32^ ^]^ In addition, hydrogens are prone to diffuse along an empty channel within the lattice of correlated oxides, rendering the crystal facet anisotropy in hydrogen‐related electronic phase modulations.^[^ ^33^ ^]^ Delicately designing the material microstructure is poised to create unblocked conduits for hydrogen diffusion, allowing an enhanced hydrogen diffusivity. From a microscopic perspective, the ability to control the hydrogen spatial distribution offers a powerful tool for precisely tailoring hydrogen‐related electronic state evolution within an electron‐correlated system. Establishing the relationship between microscopic hydrogen distribution and macroscopic electronic phase modulation not only deepens the understanding of how the protons behave in phase transition but also drives forward protonic device applications.

**Figure 1:**
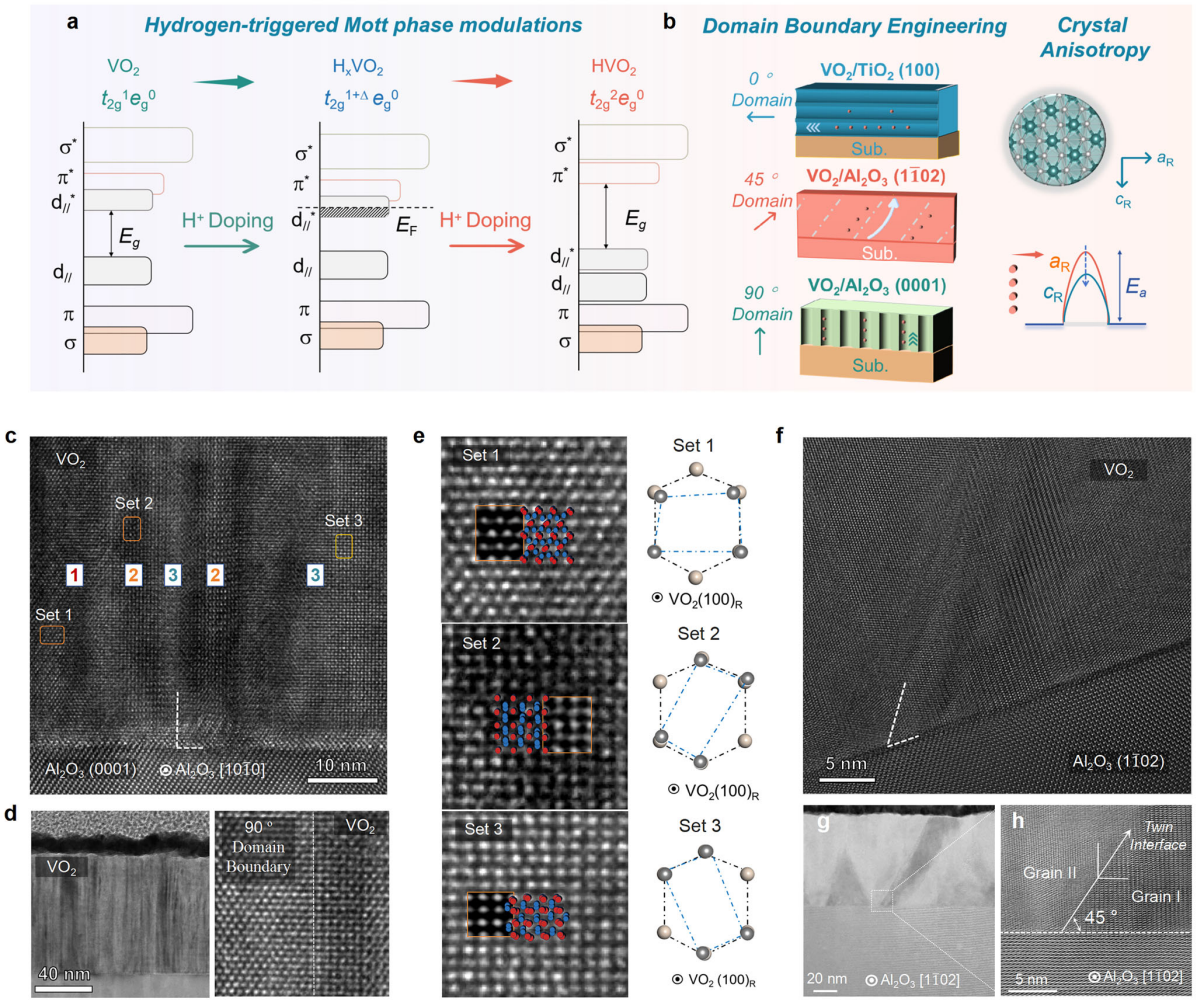
Artificial design in the material microstructure of correlated VO_2_. a) Schematic illustration of the hydrogen‐related Mott phase modulations within VO_2_ system. b) Schematic of artificially engineering the domain boundary and crystal orientation of VO_2_. c) High‐resolution transmission electron microscopy (HRTEM) images for as‐deposited VO_2_/Al_2_O_3_ (0001) heterostructure. d) Visualization of the vertically‐aligned domain boundary in VO_2_ deposited on the *c*‐plane Al_2_O_3_ substrate. e) HRTEM images for three sets of equivalent atomic arrangements of VO_2_ grown on the *c*‐plane Al_2_O_3_ substrate. f) HRTEM images for as‐deposited VO_2_/Al_2_O_3_ (1$\bar{1}$02) heterostructure. g) Low‐magnification and h, high‐magnification images for visualizing the 45°‐tilted domain boundary in VO_2_/Al_2_O_3_ (1$\bar{1}$02) bilayer using the HRTEM analysis.

Here, we identified VO_2_, an electron‐correlated system exceptionally responsive to external stimuli, as an ideal platform for adjusting proton evolution through delicately designing material microstructure. Conventionally, VO_2_ undergoes an abrupt insulator‐metal transition that is driven by a critical temperature of 341K, accompanied by a symmetry‐lowering structural transformation from low‐temperature monoclinic phase to high‐temperature rutile phase.^[^ ^34^ ^]^ Beyond thermally‐driven phase transition, hydrogenation triggers a sequential insulator‐metal‐highly insulator Mott phase transition in the VO_2_ system.^[^ ^14^, ^24^ ^]^ Introducing the electron carriers to partially occupy the low‐energy *d*_//_^*^ orbital through proton evolution triggers the metallization of VO_2_. By strong contrast, with excessive hydrogenation, the complete filling in the *d*_//_^*^ orbital of VO_2_ instead opens a wider bandgap between *d*_//_^*^ and *π* ^*^ orbitals, leading to the electron localization. This breakthrough enables the possibility in designing electronic device applications using the proton evolution, but realizing high‐speed operation remains a technical challenge due to the inherently slow bulk diffusion kinetics. To facilitate proton evolution, it is highly desirable to delicately design the material microstructure for building up an unobstructed conduit for hydrogen diffusion, which can significantly reduce the hydrogen diffusion barrier (**Figure** **1**b). In this work, the artificial design of material microstructure is identified as an effective pathway to expedite electronic phase transition via establishing an unobstructed freeway for hydrogen diffusion. In particular, an abnormal uphill hydrogen distribution, opposite to the hydrogen concentration gradient, was observed for VO_2_ with a pre‐existing unobstructed conduit, indicating a close relationship between microscopic hydrogen distribution and macroscopic phase transition. This work highlights the robust capability of manipulation on the ionic evolution of VO_2_ by taking advantage of the microstructure engineering, offering an promising pathway for designing high‐speed protonic devices.

## 2 Results and Discussion

To address the above central concept, the differently oriented rutile TiO_2_ and hexagonal Al_2_O_3_ are intentionally selected as an epitaxial template for designing the domain boundary configuration and crystal orientation of rutile VO_2_ films. Noting a similar *a*‐axis lattice constant and identical rutile structure between VO_2_ and TiO_2_, the rutile‐on‐rutile coherent epitaxial growth of VO_2_ film deposited on the TiO_2_ (100) substrate is expected to engender the horizontally‐aligned domain boundary in VO_2_ film that tightly conforms to the substrate orientation. The coherent epitaxy in VO_2_/TiO_2_ heterostructure starkly differs from the situation of rutile VO_2_ film grown on hexagonal Al_2_O_3_ substrate, in which case the symmetry mismatch is expected to induce potential lattice rotation and twinning tilt of VO_2_, incurring a domain‐matching epitaxial growth. This understanding is further identified by the high‐resolution transmission electron microscopy (HRTEM) in Figure 1c, where the vertically‐aligned domain boundaries are visualized in as‐deposited VO_2_/Al_2_O_3_ (0001) (i.e., *c*‐plane Al_2_O_3_) heterostructure (Figure 1d). The symmetry mismatch between hexagonal Al_2_O_3_ and rutile VO_2_, coupled with the *β* angular deviation of VO_2_ (e.g., 122.6°) from the Al_2_O_3_ (e.g., 120°), leads to three equivalent sets of twin variants in VO_2_ film (Figure 1e). It is found that the atomic arrangement in set 1 resembles the (001) plane of rutile VO_2_, while the zigzag V‐V chain in set 2 (e.g., [011]_R_ domain) related to the dimerization of VO_2_ results in a lowering of crystal symmetry, in comparison with the [01$\bar{1}$]_R_ domain of VO_2_ in set 3 (Figure S1, Supporting Information).^[^ ^35^ ^]^ By contrast, a 45 °‐tilted twin boundary relative to the heterointerface was observed for as‐deposited VO_2_/Al_2_O_3_ (1$\bar{1}$02) (i.e., *r*‐plane Al_2_O_3_) heterostructure in Figure 1f–h. This phenomenon is ascribed to two equivalent (200)‐faceted and ($\bar{2}$11)‐faceted domains existed in the VO_2_ film grown on the *r*‐plane Al_2_O_3_ substrate, in which the angle between (200) and ($\bar{2}$11) planes is estimated to be 44.8 °, aligning well with the observed twinning tilt of ≈ 45 °. Given that the VO_2_ films deposited on TiO_2_ (100), Al_2_O_3_ (0001) and Al_2_O_3_ (1$\bar{1}$02) substrates are strain‐relaxed,^[^ ^36^, ^37^ ^]^ we can rule out the interfacial strain as a primary factor modulating the proton evolution. In addition, the relatively smooth surface roughness for as‐deposited VO_2_ films is characterized by using the atomic force microscope in Figure S2 (Supporting Information). Consequently, the artificial design in the domain boundary configuration of VO_2_ with diverse orientations relative to the heterointerface, ranging from 0° to 90°, is realized through elaborately selecting as‐used epitaxial templates. Such precise control over the domain boundary configuration is expected to effectively tailor the proton evolution, in which an inclined or vertical domain boundary configuration acts as a freeway for promoting hydrogen diffusion via ionic interactions between hydrogens and extended planar defects. Furthermore, a 45°‐tilted oxygen vacancy channel was previously demonstrated to more effectively accelerate the ionic evolution in brownmillerite SrFeO_2.5_ compared with the 90 °‐tilted one, thus enhancing the catalytic reactions^[^ ^32^ ^]^ or resistive switching.^[^ ^38^ ^]^

**Figure 2:**
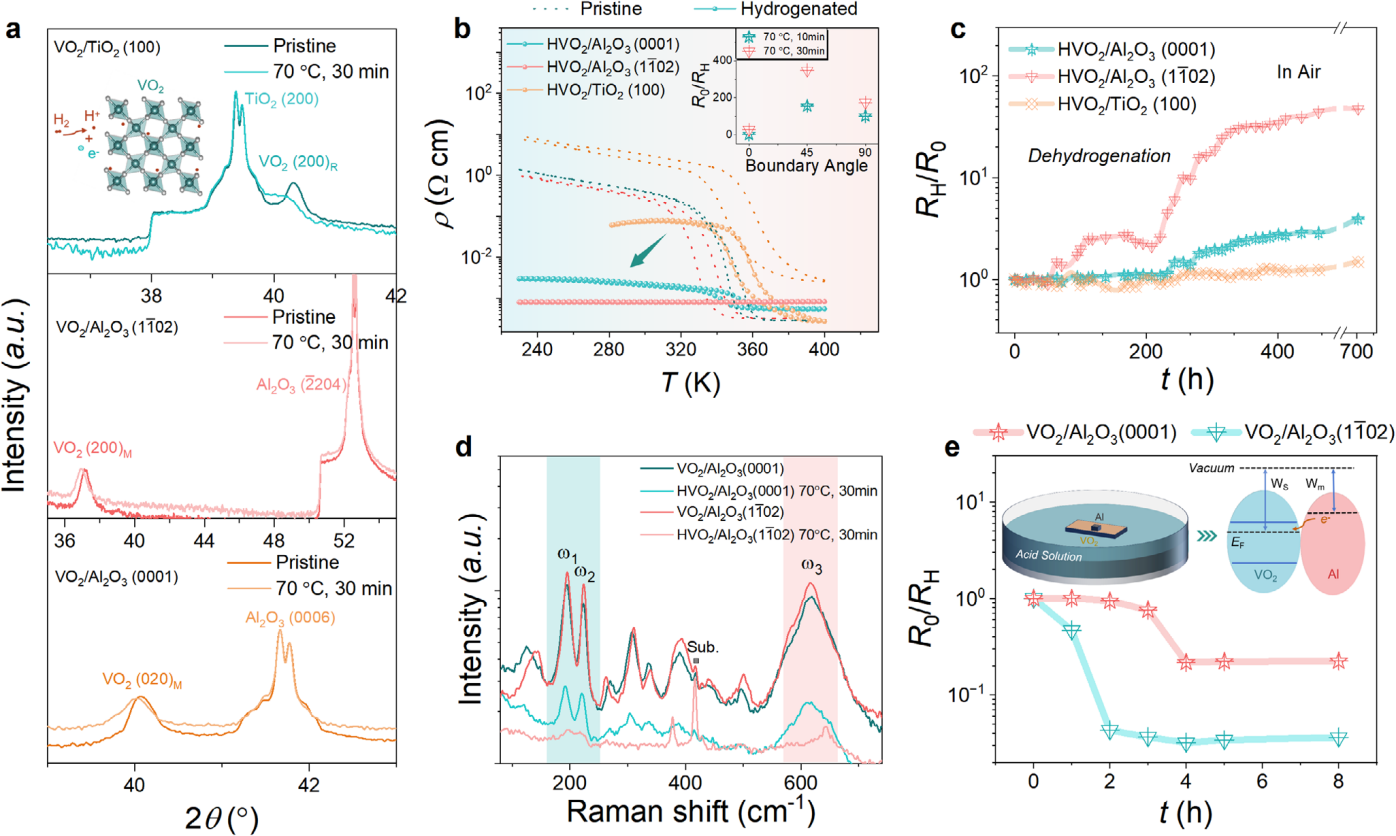
Hydrogen‐related structural evolution and electronic phase transition. a) X‐ray diffraction (XRD) patterns compared for VO_2_ films under microstructure engineering through hydrogenation. b) Temperature dependence of material resistivity (*ρ*‐*T*) as measured for VO_2_ under microstructure engineering as hydrogenated at 70 °C for 30 min, while hydrogen‐associated variation in the material resistivity (*R*_0_/*R*_H_) is shown in the inset. c) Dehydrogenation process as compared for VO_2_ under microstructure engineering via exposing to the air. d) Raman spectra as compared for VO_2_ deposited on the (0001) and (1$\bar{1}$02)‐oriented Al_2_O_3_ substrates before and after hydrogenation. e) Temporal evolution of the magnitude of *R*_0_/*R*_H_ compared for VO_2_ films deposited on the *c*‐plane and *r*‐plane Al_2_O_3_ substrates, as hydrogenated using acid solution strategy.

On this basis, a platinum (Pt)‐assisted hydrogen spillover strategy was exploited for achieving the hydrogen ions intercalation into the lattice of VO_2_. The noble metal Pt as a catalyst can substantially reduce the energy barrier for dissociating the hydrogen molecules derived from the H_2_/Ar forming gas into the protons and electrons at the triple phase boundary, triggering a catalytic reaction associated with H_2_ (g)→H^+^+e^−^.^[^ ^10^, ^39^ ^]^ To compare the hydrogenation kinetics of VO_2_ under microstructure design, a fairly mild hydrogenation condition (e.g., 70 °C, 30 min) was herein employed, in comparison with the previous reports,^[^ ^9^, ^17^, ^24^ ^]^ which simultaneously avoids the formation of potential oxygen defects. From the perspective of structural evolution, performing the hydrogenation results in the leftward shift of the characteristic diffraction peak of VO_2_ in their X‐ray diffraction (XRD) patterns, regardless of selected epitaxial templates (**Figure** **2**a). Such structural evolution in VO_2_ reveals an *out‐of‐plane* lattice expansion through hydrogenation, primarily ascribed to O─H interactions, which is more pronounced for VO_2_/Al_2_O_3_ (1$\bar{1}$02) heterostructure (Table S1, Supporting Information). It is worthy to note that the grown VO_2_ films on the (0001)‐oriented Al_2_O_3_ and (100)‐oriented TiO_2_ substrates are (010)_M_‐faceted and (100)_R_‐faceted crystal orientations, respectively, while in contrast the *r*‐plane Al_2_O_3_ template can induce the preferential growth of VO_2_ toward (100)_M_ orientation, equivalent to the *c*_R_ orientation. It is widely reported that a lower energy barrier for hydrogen diffusion can be achieved along the [001]_R_ channel of VO_2_ compared to the [100]_R_ channel, owing to an empty channel along the *c*_R_ direction as formed by the chains of edge‐sharing VO_6_ octahedra.^[^ ^40^, ^41^ ^]^ Therefore, the *r*‐plane Al_2_O_3_ template, which can simultaneously induce a *c*_R_‐faceted crystal orientation and inclined domain boundary configuration in VO_2_ film, dramatically reduces the hydrogen diffusion barrier (Table S2, Supporting Information).

The effective regulation in hydrogen‐related insulator‐metal transition through microstructure design is confirmed by comparing the temperature dependence of material resistivity in Figure 2b. With a mild hydrogenation at 70 °C for 30 min, the VO_2_/TiO_2_ (100) heterostructure still retains a pronounced thermally‐driven phase transition, akin to the pristine state. By strong contrast, the electronic phase transition of VO_2_/Al_2_O_3_ (0001) bilayer is dramatically depressed upon hydrogenation, resulting in a transition sharpness (*ρ*_Insul._/*ρ*_Metal._) below 10^1^. Of particular note is the complete metallization for the VO_2_ film deposited on *r*‐plane Al_2_O_3_ substrate, identifying an accelerated hydrogenation kinetics through microstructure design. The inclined boundary configuration and preferential *c*_R_‐faceted orientation as simultaneously realized in the VO_2_/Al_2_O_3_ (1$\bar{1}$02) heterostructure facilitates the proton evolution via lowering the diffusion barrier of hydrogen, thereby enhancing resultant electronic phase modulation (Figure S3a, Supporting Information). This understanding is further confirmed by comparing the hydrogen‐related resistive regulation (e.g., *R*_0_/*R*_H_) of VO_2_ under microstructure engineering, in which a more pronounced *R*_0_/*R*_H_ is realized in VO_2_/Al_2_O_3_ (1$\bar{1}$02) bilayer as shown in the inset of Figure 2b. The above regulation on the proton evolution through microstructure design is reproducible (Figures S3b,c and S4 and Tables S3 and S4, Supporting Information) and analogously observed in VO_2_ hydrogenated at 70 °C for 10 min (Figure S5, Supporting Information). Further consistency is verified by the respective dehydrogenation process in Figure 2c via exposing hydrogenated VO_2_ to the air, during which the intercalated hydrogens within VO_2_ tend to be dragged out owing to a low crystal trapping potential and ultrahigh chemical diffusivity. Similarly, corresponding dehydrogenation process is effectively expedited by using artificial microstructure design, evidenced by a reduced onset time for triggering resistive recovery and an elevated magnitude of *R*_H_/*R*_0_ in VO_2_/Al_2_O_3_ (1$\bar{1}$02) bilayer. It is worthy to note that hydrogen‐related phase transition and structural evolution within VO_2_ system are highly reversible when annealed in the air at 70 °C for 30 min, in alignment with the prior reports (Figures S6 and S7, Supporting Information).^[^ ^2^ ^]^

In addition, hydrogen‐associated structural transformation is further determined using the Raman spectra in Figure 2d, where the Raman peaks at 194, 223, and 612 cm^−1^ can be taken as direct indicators of structural transformation in VO_2_ system. The Raman peaks located at 194 cm^−1^ (ω1) and 223 cm^−1^ (ω2) correspond to the interaction of V‐V dimers, while the Raman peak at 612 cm^−1^ (ω3) reflects the difference in the V─O bond length.^[^ ^2^ ^]^ Such characteristic Raman peaks are almost suppressed for VO_2_/Al_2_O_3_ (1$\bar{1}$02) heterostructure through hydrogenation, revealing the conversion to a rutile‐like crystal structure, accompanied by the suppression of V‐V dimers. This observation differs from the case of VO_2_/Al_2_O_3_ (0001) bilayer, in which situation the above Raman peaks (e.g., ω1 and ω2 peaks) still remain detectable upon hydrogenation, signifying remnant monoclinic phase that cannot transit to rutile phase. Without loss of generality, a non‐catalytic hydrogenation strategy leveraging the electron‐proton co‐doping was exploited to generalize the artificial microstructure design strategy for adjusting the proton evolution.^[^ ^42^ ^]^ In this scenario, the Fermi level difference between the low work‐function metal (e.g., Al) and VO_2_ induces a spontaneous electron transfer from Al particle to VO_2_ film, which attracts the protons from the acid solution to be intercalated into the lattice of VO_2_, realizing the hydrogenation (Figure S8, Supporting Information).^[^ ^42^ ^]^ Hydrogen‐associated structural evolution of VO_2_ using acid solution strategy is demonstrated by XRD spectra (Figure S9, Supporting Information). More importantly, the onset time for triggering the resistive reduction in VO_2_/Al_2_O_3_ (1$\bar{1}$02) heterostructure through hydrogenation is significantly lower than the one grown on widely‐used (0001)‐oriented Al_2_O_3_ substrate by 2–3 times,^[^ ^24^, ^25^, ^27^, ^42^, ^43^ ^]^ indicative of an accelerated kinetics of proton evolution (Figure 2e). Meanwhile, the hydrogen‐related resistive switching (e.g., *R*_0_/*R*_H_) achievable in VO_2_/Al_2_O_3_ (1$\bar{1}$02) hybrid largely exceeds the one for VO_2_ deposited on the *c*‐plane Al_2_O_3_ by almost one order of magnitude. Thereby, establishing an unobstructed conduit through artificially designing the material microstructure kinetically promotes the electronic phase modulations in correlated VO_2_ system.

**Figure 3:**
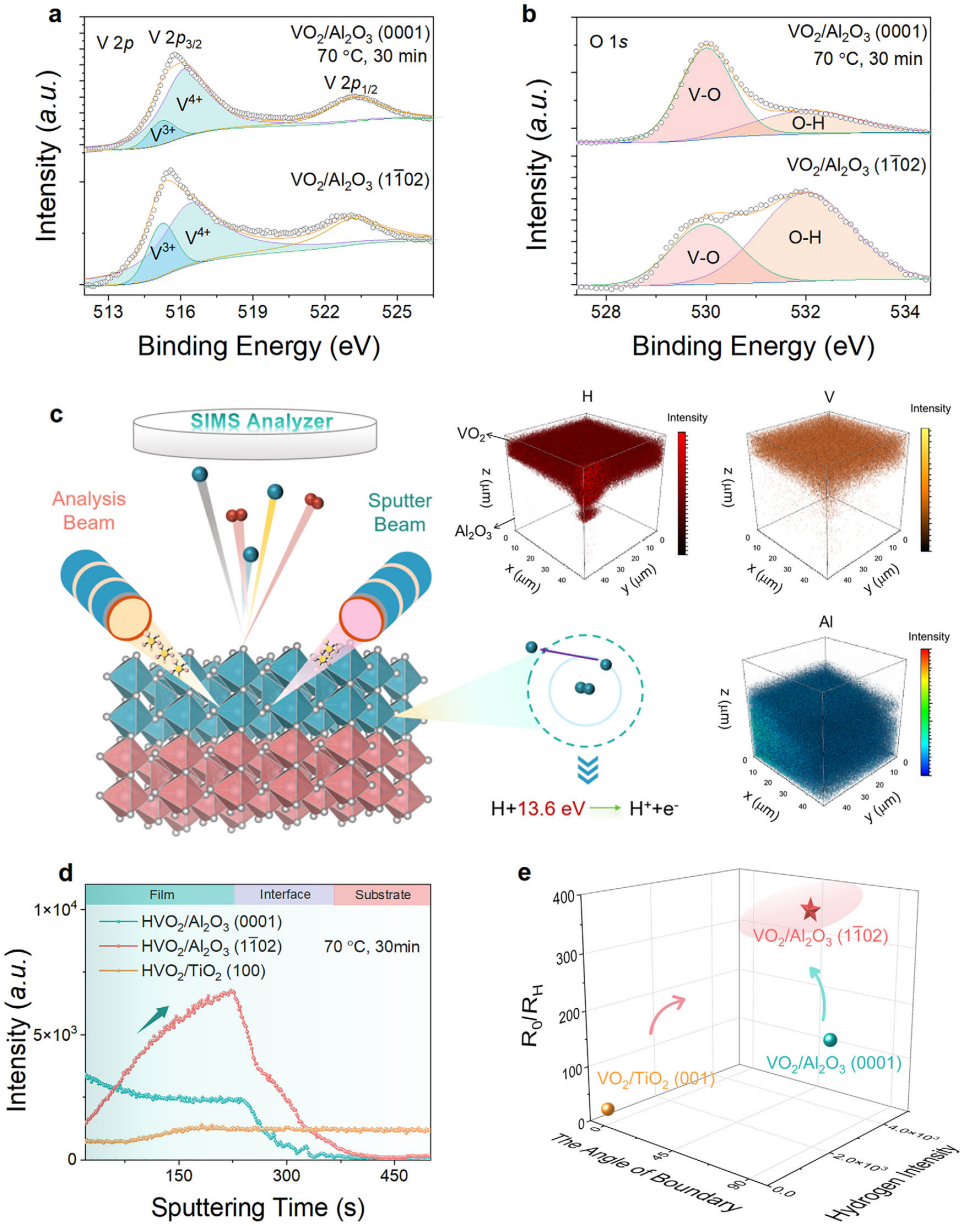
Hydrogenation kinetics and chemical environment for VO_2_. a,b) X‐ray photoelectron spectra (XPS) spectra for the core levels of (a) vanadium and (b) oxygen of hydrogenated VO_2_ under microstructure engineering. c) Schematic of the working principle of time‐of‐flight secondary ion mass spectrometry (ToF‐SIMS) and 3D ToF‐SIMS element maps for hydrogenated VO_2_/Al_2_O_3_ (1$\bar{1}$02). d) Depth‐profile of the hydrogen concentration compared for VO_2_ under microstructure engineering upon identical hydrogenation conditions. e) The magnitude of *R*_0_/*R*_H_ plotted as a function of hydrogen concentration as compared for VO_2_ under microstructure engineering.

To clarify the variations in the chemical environment of VO_2_ under hydrogenation, X‐ray photoelectron spectra (XPS) analysis was performed, as the V 2*p* core‐level spectrum shown in **Figure** **3**a. It is found that the valence state of vanadium is reduced from V^4+^ to V^(4−δ)+^ upon hydrogenation, particularly for the VO_2_/Al_2_O_3_ (1$\bar{1}$02) hybrid. In addition, performing the hydrogenation elevates the relative intensity of the O─H interaction (e.g., ≈532 eV) for VO_2_ with respect to the V─O interaction (e.g., ≈530 eV) (Figure 3b).^[^ ^16^ ^]^ The O─H interaction as formed in hydrogenated VO_2_ indicates that the intercalated hydrogens acting as interstitial defects readily bond with the lattice oxygen, forming the O─H weak bonds, in accordance with previous XRD results. Furthermore, a more pronounced elevation in the O─H interaction was observed for hydrogenated VO_2_/Al_2_O_3_ (1$\bar{1}$02) heterostructure in comparison with the VO_2_/Al_2_O_3_ (0001) (Figure S10, Supporting Information), which further demonstrates an overwhelming advantage of a freeway in promoting proton evolution created by 45°‐tilted twin boundary and preferential *c*_R_ orientation.

In order to provide more direct evidence for hydrogen spatial distribution within the lattice of VO_2_, time‐of‐flight secondary ion mass spectrometry (ToF‐SIMS) analysis was performed to semiquantitatively characterize the depth profiles of hydrogen concentration. ToF‐SIMS analysis as a destructive technique mainly utilized an ion beam (e.g., Ar_n_^+^ and Cs^+^) to sputter through the hydrogenated VO_2_ film, ejecting the molecular ions that can be further analyzed using a mass spectrometer (Figure 3c).^[^ ^44^ ^]^ In addition, the change in the intensity of molecular species over sputtering time provides a depth‐resolved understanding of incorporated hydrogen concentration, which identifies microscopic hydrogen distribution within the lattice of VO_2_. In VO_2_ films deposited on *c*‐plane and *r*‐plane Al_2_O_3_ substrates, the presence of hydrogen is evident over the film region via 3D ToF‐SIMS element maps (Figure 3c; Figure S11, Supporting Information), contrasting with VO_2_/TiO_2_ (100) bilayer where no significant H signal discrepancy exists between film and substrate regions (Figure S12, Supporting Information).

The kinetic acceleration in hydrogen diffusion through the artificial design of material microstructure is indirectly indicated by comparing the depth profile of hydrogen concentration, as performed using ToF‐SIMS analysis in Figure 3d. With a fairly mild hydrogenation (e.g., at 70 °C for 30 min), a significantly higher hydrogen intensity is observed for the grown VO_2_ film in comparison with the *c*‐plane Al_2_O_3_ substrate, unveiling an effective hydrogen incorporation, which differs from VO_2_/TiO_2_ (100) hybrid with a similar H intensity over the entire heterostructure region. The vertical domain boundary configuration in VO_2_/Al_2_O_3_ (0001) bilayer promotes the hydrogen diffusion, compared with the VO_2_/TiO_2_ (100) with horizontally‐aligned domain boundary, in agreement with a previous study (Figures S13 and S14, Supporting Information).^[^ ^32^, ^43^ ^]^ Surprisingly, an uphill hydrogen distribution is observed for VO_2_/Al_2_O_3_ (1$\bar{1}$02) bilayer, where the progressive increase in hydrogen concentration with diffusion length fundamentally deviates from basic Fick's law of diffusion (Figure S15, Supporting Information). This phenomenon stands stark contrast with conventional understanding of hydrogen diffusion, which is driven by the hydrogen concentration gradient as described by the Fick's law of diffusion. Such the anomalous uphill hydrogen diffusion realized in the VO_2_/Al_2_O_3_ (1$\bar{1}$02) bilayer is associated with the pre‐existing unobstructed freeway, in favor of hydrogen diffusion. Notably, the initial vanadium signal enhancement at low sputtering times is associated with the existence of surface contaminants on the sample surface, such as hydrocarbons, preferentially removed during early sputtering. Therefore, spatial hydrogen distributions are quantified using the stabilized hydrogen signal regime (e.g., from 20 to 500 s) beyond this initial stage. The averaged hydrogen concentration achievable in VO_2_/Al_2_O_3_ (1$\bar{1}$02) bilayer also exceeds the one for widely‐reported VO_2_/Al_2_O_3_ (0001) and VO_2_/TiO_2_ (100) heterostructures (Figure 3e; Table S5, Supporting Information).^[^ ^24^, ^25^, ^27^, ^42^, ^43^ ^]^ In addition, the discrepancy in the incorporated hydrogen concentration among the VO_2_ films, achieved under identical hydrogenation conditions, qualitatively reflects an elevation in the diffusion coefficient of hydrogen through microstructure design, according to Fick's laws of diffusion. This result further demonstrates a close correlation between hydrogen spatial distribution and macroscopic phase modulations. It is in particular worthy to note that oxygen defects as inevitably formed during the reductive annealing may synergistically contribute to the carrier delocalization of VO_2_, in which the incorporation of oxygen defects also stabilizes the metallic phase. Therefore, apart from the incorporated hydrogens, the possible emergence of oxygen vacancy in VO_2_ film, as well as the variations in bonding environment through hydrogenation, cannot be neglected. In terms of protonic device application, such the uphill hydrogen distribution realized in VO_2_ through microstructure design is also poised to mitigate the need for significant energy input typically associated with hydrogen supply. Future protonic devices are expected to harness artificial microstructure engineering of VO_2_ to accelerate hydrogenation kinetics and resultant electronic state evolution, boosting high‐speed correlated electronics. Nevertheless, inhomogeneous hydrogen distribution within VO_2_ films through artificial microstructure design, together with incompatibility with conventional CMOS processes, remains a major impediment to industrialized implementation. In particular, brownmillerite transitional metal oxides with tunable oxygen vacancy channel (e.g., SrFeO_2.5_, SrCoO_2.5,_ and CaFeO_2.5_) offer a fertile ground to tailor the ionic evolution via using the artificial design of material microstructure.

**Figure 4:**
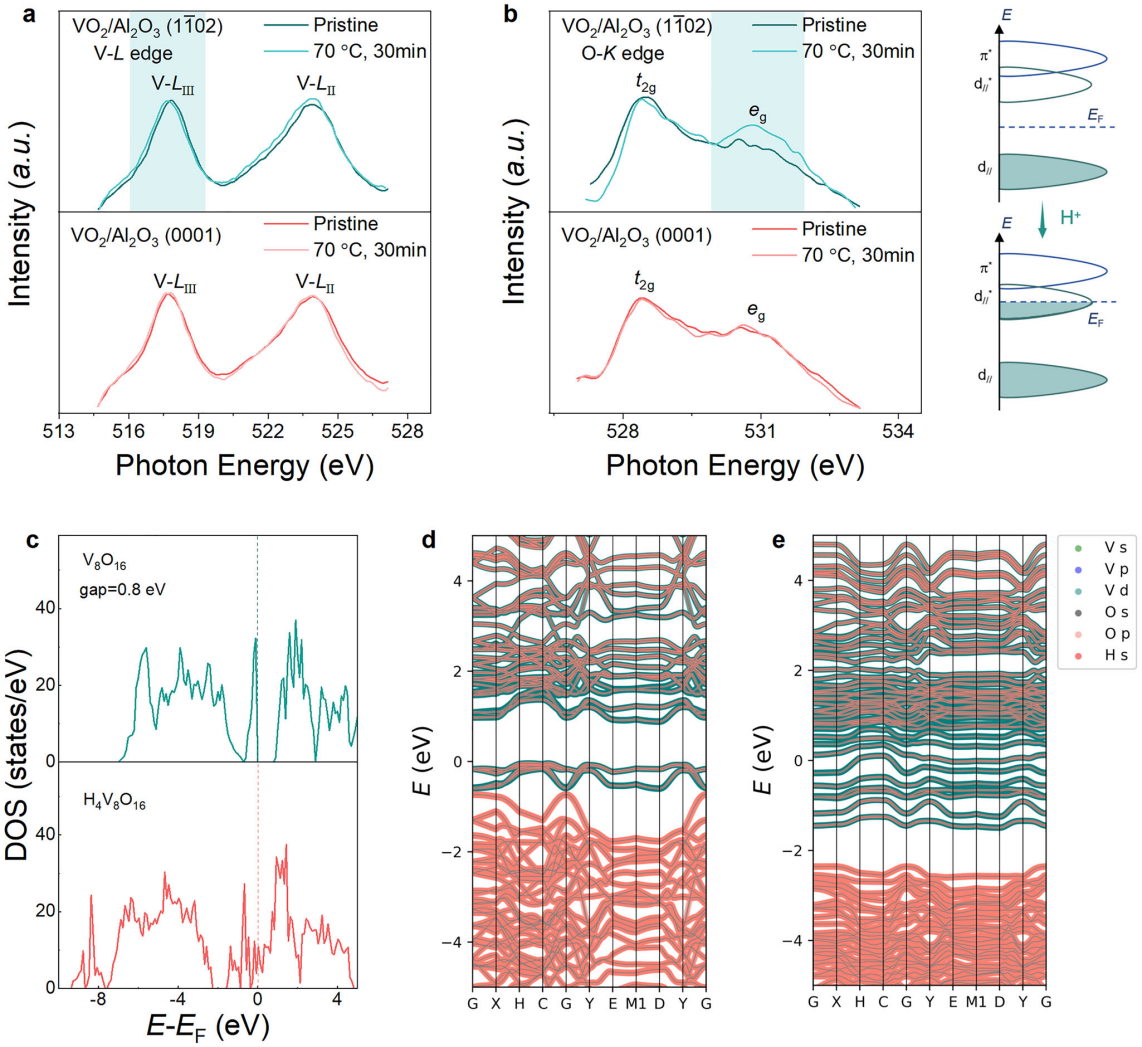
Band structure for hydrogenated VO_2_. a,b) Soft X‐ray absorption spectroscopy (sXAS) for the (a) V‐*L* edge and (b) O‐*K* edge of VO_2_/Al_2_O_3_ (1$\bar{1}$02) bilayer through hydrogenation, in comparison with the VO_2_/Al_2_O_3_ (0001) heterostructure. The changes in the electronic orbital configuration of VO_2_ through hydrogenated are schematically shown in the right. c) Calculated density of states (DOS) of VO_2_ film c, before and d, after hydrogenation. d,e) Calculated fat band structure for (d) pristine VO_2_, and (e) H_4_V_8_O_16_.

To probe the physical origin driving hydrogen‐related electronic phase modulations, the electronic band structure of VO_2_ is characterized using the soft X‐ray absorption spectroscopy (sXAS) technique and density functional theory (DFT) calculations in **Figure** **4**. The V‐*L* edge spectrum associated with the V 2*p* → 3*d* transition is widely recognized to effectively reflect the variations in the vanadium valence state.^[^ ^42^ ^]^ Performing the hydrogenation results in the leftward shift of both V‐*L*_III_ and V‐*L*_II_ peaks, unraveling the reduction in the valence state of vanadium (Figure 4a), while such the hydrogen‐related red shift is more pronounced for VO_2_/Al_2_O_3_ (1$\bar{1}$02) heterostructure, reminiscent of that found in respective XPS spectra. Noting empty O‐2*p* states and the hybridization between V‐3*d* and O‐2*p* orbitals, the unoccupied density of states in the conduction band of VO_2_ can be reflected by the O 1*s* spectrum, in which the relative variation in the first (second) peak intensity qualitatively represents the electron occupation in the *t*_2g_ (*e*_g_) band of VO_2_.^[^ ^9^ ^]^ Upon hydrogenation, the reduction in the spectral weight of the first peak in O 1*s* core‐level spectrum with respect to the second peak unveils the band filling in the low‐energy *t*_2g_ band of VO_2_, which is formed by *d*_xz_, *d*_yz,_ and $d_{x^{2}-y^{2}}$ orbitals (Figure 4b). Compared with the VO_2_/Al_2_O_3_ (0001) hybrid, a more abrupt reduction in the ratio of *t*_2g_/*e*_g_ peak observed for VO_2_/Al_2_O_3_ (1$\bar{1}$02) through hydrogenation is ascribed to the kinetically accelerated proton evolution that introduces more extensive electron doping in the *t*_2g_ orbital of VO_2_. Analogous variations in the sXAS spectra are realized in VO_2_ film as hydrogenated at 70 °C for 10 min (Figure S16, Supporting Information). On the basis of electronic band structure, the doped electron carriers prefer to occupy the low‐energy *d*_//_^*^ orbital of VO_2_ to reconfigure the band structure, driving a collective metallization.

The underneath mechanism governing the hydrogen‐associated insulator‐metal transition within VO_2_ is more clearly clarified via analyzing the electronic density of states (DOS) using DFT calculations (Figure 4c; Figure S17, Supporting Information). It is found that a bandgap of ≈ 0.8 eV is observed for pristine VO_2_, in accordance with its correlated electronic ground state (Figure 4d). However, introducing hydrogens into the lattice of VO_2_ (e.g., H_4_V_8_O_16_ with 0.5 H per u.c.) renders the metallization of VO_2_, evidenced by the finite DOS near the *E*_F_ (Figure 4e). Furthermore, the electron carriers provided by hydrogenation are prone to occupy the V‐3*d* orbital, according to DFT calculations. By virtue of DFT calculations and sXAS analysis, the physical origin underlying hydrogen‐associated electronic phase transition is identified as band‐filling‐mediated orbital reconfiguration. Moreover, utilizing the ultraviolet photoelectron spectroscopy (UPS) technique, the work function of VO_2_ is demonstrated to be reduced through hydrogenation, e.g., from 5.12–5.85  to 4.69–4.74 eV, owing to hydrogen‐associated electron doping (Figure S18, Supporting Information).

## 3 Conclusion

In summary, we identified a feasible strategy to tailor hydrogen‐associated IMT in a correlated VO_2_ system through establishing an unobstructed freeway for promoting hydrogen diffusion using artificial microstructure design. The domain‐matching epitaxy growth of VO_2_ film on Al_2_O_3_ substrate resulting from the symmetry mismatch, along with the rutile‐on‐rutile coherent growth in VO_2_/TiO_2_ bilayer, serves as a promising platform for delicately designing the VO_2_ microstructure. By leveraging a 45°‐tilted boundary configuration and *c*_R_‐faceted crystal orientation, simultaneously induced by *r*‐plane Al_2_O_3_ template, the diffusion barrier of hydrogens is extensively reduced, leading to an unusual uphill hydrogen diffusion behavior that violates Fick's law of diffusion. As a result, hydrogen‐related electronic phase modulation is achievable in VO_2_/Al_2_O_3_ (1$\bar{1}$02) hybrid is significantly enhanced compared to the one grown on the widely‐used *c*‐plane Al_2_O_3_ substrate,^[^ ^24^, ^25^, ^27^, ^42^, ^43^ ^]^ with resistive switching improved by an order of magnitude and switching speed expedited by 2–3 times. Our findings provide compelling evidences for the close correlation between hydrogen spatial distribution and macroscopic filling‐controlled Mott phase transitions, suggesting a promising pathway for precisely tailoring hydrogenated electronic state. Furthermore, we showcase the robust capability of manipulating ionic evolution in correlated oxide systems by virtue of artificial microstructure design, offering a proof‐of‐principle for designing advanced protonic devices.

## 4 Experimental Section

### Fabrication of the Grown VO_2_ Heterostructures

The VO_2_ films were deposited on the single crystalline (100)‐oriented TiO_2_ and (0001) or (1$\bar{1}$02)‐oriented Al_2_O_3_ substrates using the laser molecular beam epitaxy (LMBE) technique. The deposition temperature, the oxygen pressure, the target‐substrate distance and the laser fluence were optimized as 500 °C, 1.5 Pa, 45 mm, and 1.0 J cm^2^, respectively. Afterward, the as‐deposited VO_2_ films were naturally cooled down to room temperature under identical oxygen partial pressure. Before the hydrogenation, the 20 nm‐thick platinum dots were sputtered into the surface of the grown VO_2_ films via exploiting the magnetron sputtering technique. Finally, based on the hydrogen spillover strategy, as‐made Pt/VO_2_ heterostructures were annealed in a 5% H_2_/Ar forming gas for effectively realizing hydrogenation. Additionally, a metal‐assisted acid solution strategy was also employed to hydrogenate the VO_2_ films, in which a 1 × 1 × 1 mm^3^ Al particle was placed onto the surface of VO_2_ films immersed in a dilute sulfuric acid solution.

### Material Characterizations

The crystal structures of the grown VO_2_ films were probed via using the X‐ray diffraction (XRD) (Rigaku, Ultima IV) and Raman spectra (HORIBA, HR Evolution). High‐resolution transmission electron microscopy (HRTEM) (JEOL, JEM F200; JEM, ARM300F) was also performed to characterize the crystal structure of VO_2_ films that were first fabricated via using the focused ion beam (FIB) (FEI, G4 UX). The variations in the chemical environment of hydrogenated VO_2_ were assessed by using the X‐ray photoelectron spectroscopy (XPS) technique (Thermo, K‐Alpha X). The electronic structure of VO_2_ films was further explored through soft X‐ray absorption spectroscopy (sXAS) analysis, as conducted at the Shanghai Synchrotron Radiation Facility (SSRF) on beamline BL08U1A. Temperature dependences of resistivity for the deposited VO_2_ films were measured by using a commercial physical property measurement system (PPMS) (Quantum design), meanwhile the room‐temperature resistance was determined by using a Keithley 2400 system. The depth profile of hydrogen is examined by using the time‐of‐flight secondary ion mass spectrometry (ToF‐SIMS) technique (ION‐TOF GmbH, TOF.SIMS 5).

### First‐Principles Calculations

First‐principles calculations were conducted using the projector augmented wave (PAW) method within the QUANTUM ESPRESSO framework. To model the hydrogenated VO_2_ (H_4_V_8_O_16_), a 2 × 1 × 1 supercell was expanded along the *a*‐axis, incorporating two hydrogen atoms per unit cell. The computational setup included a plane‐wave kinetic energy cutoff of 90 Ry and a 5 × 11 × 9 Monkhorst‐Pack k‐point mesh for sampling the Brillouin zone. Electron correlation effects in the V‐3*d* orbitals were accounted for via the GGA+U method,^[^ ^45^ ^]^ with a Hubbard *U* value of 3.32 eV applied to the V‐3*d* states owing to the strong electron correlations. Structural optimization was achieved by relaxing atomic positions until the inter‐atomic forces were reduced below 10^−3^ Ry/Bohr. Self‐consistent field calculations were performed with an energy convergence criterion of 10^−12^ Ry to ensure high numerical accuracy. Finally, the hydrogen‐associated electronic band structure was computed based on the optimized geometry, facilitating a detailed investigation into the changes in electronic states induced by hydrogenation.

## Conflict of Interest

The authors declare no conflict of interest.

## Author Contributions

X.Z., X.Y., and W.L. contributed equally to this work. X.Z. conceived this study, and lead the project; X.Z., G.Z., and X.X. supervised the study; X.Z. planned for the experiment, and analyzed the results; X.Y., J.J., and X.Q. grew VO_2_ films, and carried out the transport measurements under the supervision of X.Z. and X.X.; W.L. performed the DFT calculations assisted by C.Y. and Z.Y.; J.G. and L.L. performed the TEM analysis; X.Z., X.X., G.Z., and H.J. provided the supports in the sample prepares and transport measurements; X.Z. wrote the paper with contributions from all authors; All authors discussed the results and commented on the final manuscript.

## Supporting information



Supporting Information

## Data Availability

The data that support the findings of this study are available from the corresponding author upon reasonable request.
