# Supplementary material for: Manipulating the Hydrogen‐Associated Insulator‐Metal Transition Through Artificial Microstructure Engineering
Source: Adv Sci (Weinh). 2025 Oct 13;13(1):e10771. doi: 10.1002/advs.202510771 (PMC12767000; doi:10.1002/advs.202510771)
Supplement: Supplementary file 1 — Supporting Information [file ADVS-13-e10771-s001.docx]

Supporting Information

**Manipulating the hydrogen-associated insulator-metal transition through artificial microstructure engineering**

*Xuanchi Zhou ^*^, Xiaohui Yao, Wentian Lu , Jinjian Guo, Jiahui Ji, Lili Lang,*

*Guowei Zhou^*^, Chunwei Yao, Xiaomei Qiao, Huihui Ji, Zhe Yuan, Xiaohong Xu ^*^*

X.C. Zhou, X.H. Yao, W.T. Lu, J.J. Guo, J.J. Ji, G.W. Zhou, C.W. Yao, X.M. Qiao, H.H. Ji, X.H. Xu

Key Laboratory of Magnetic Molecules and Magnetic Information Materials of Ministry of Education & School of Chemistry and Materials Science, Shanxi Normal University, Taiyuan, 030031, China

E-mail: [xuanchizhou@sxnu.edu.cn](mailto:xuanchizhou@sxnu.edu.cn), [zhougw@sxnu.edu.cn](mailto:zhougw@sxnu.edu.cn), [xuxh@sxnu.edu.cn](mailto:xuxh@sxnu.edu.cn)

X.C. Zhou, W.T. Lu, G.W. Zhou, H.H. Ji, X.H. Xu

Research Institute of Materials Science, Shanxi Key Laboratory of Advanced Magnetic Materials and Devices, Shanxi Normal University, Taiyuan 030031, China

L.L. Lang

National Key Laboratory of Materials for Integrated Circuits, Shanghai Institute of Microsystem and Information Technology, Chinese Academy of Sciences, Shanghai 200050, China

Z. Yuan

Interdisciplinary Center for Theoretical Physics and Information Sciences, Institute of Nanoelectronic Devices and Quantum Computing, Fudan University, Shanghai 200433, China

**This file includes:**

Supplementary Figures 1-18, Supplementary Tables 1-5, Supplementary Note 1 and Supplementary References.

**Contents**

**Figure S1. Visualization of multi-domain for VO_2_/Al_2_O_3_ (0001) heterostructure**

**Figure S2. AFM images for VO_2_ with the microstructure engineering**

**Figures S3-S4. Reproducible experiments**

**Figure S5. Regulating hydrogen-related electronic phase modulations of VO_2_ through microstructure engineering**

**Figures S6-S7. Reversibility in the proton evolution of VO_2_**

**Figure S8. Schematic of the metal-assisted acid solution hydrogenation strategy**

**Figure S9. XRD spectra for hydrogenated VO_2_ using acid solution strategy**

**Figure S10. Electron occupancy for hydrogenated VO_2_**

**Figures S11-S12. Three-dimensional element maps for hydrogenated VO_2_**

**Figures S13-S15. Depth profile of element distribution for hydrogenated VO_2_**

**Figure S16. sXAS spectra for hydrogenated VO_2_**

**Figure S17. Calculated band structure for hydrogenated VO_2_**

**Figure S18. UPS spectra for hydrogenated VO_2_**

**Table S1-S5**

**Supplementary Note 1**

**Supplementary References**


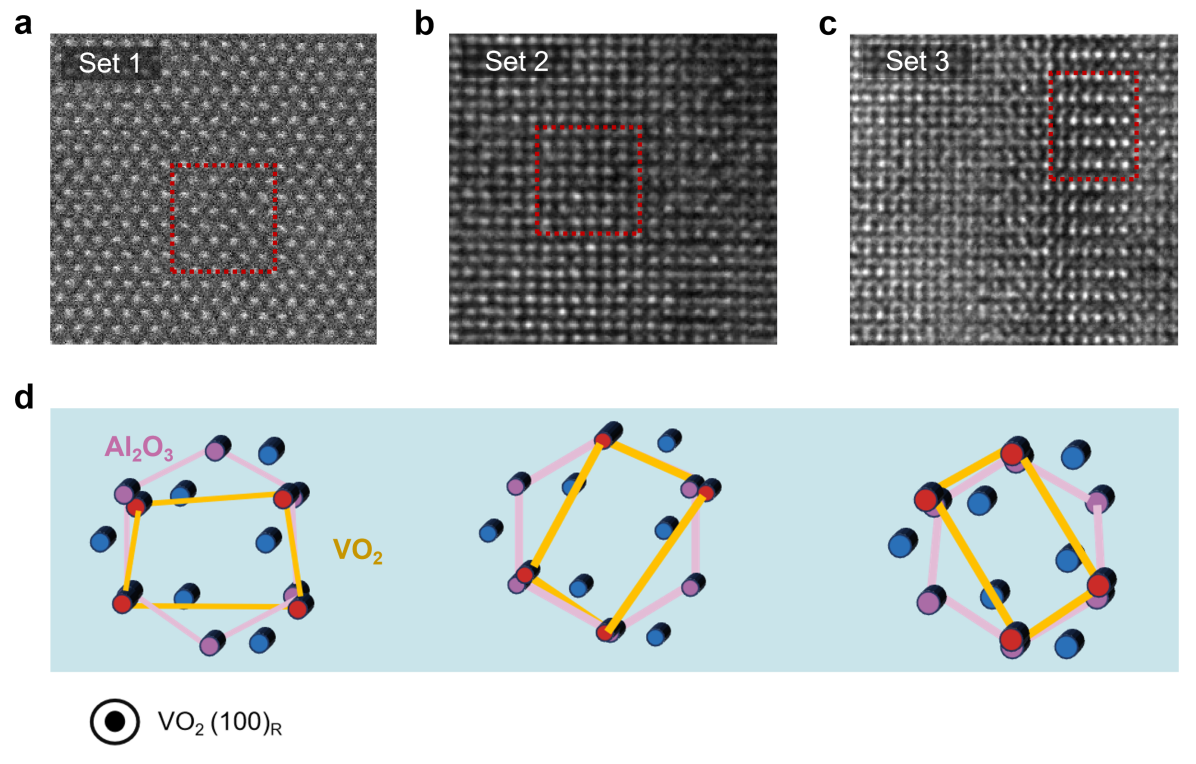


**Supplementary Figure 1.** High-resolution transmission electron microscopy (HRTEM) images for three possible **a**, [001]_R_, **b**, [011]_R_ and **c**, [01$\bar{1}$]_R_ domains of VO_2_ film deposited on the Al_2_O_3_ (0001) substrate. **d**, Schematic of potential atomic configurations for the grown VO_2_ films on the Al_2_O_3_ (0001) substrate. It is found that the symmetry mismatch between rutile VO_2_ film and hexagonal Al_2_O_3_ substrate induces such the three set of equivalent twin variants in the lattice of VO_2_, thereby resulting in the vertically-aligned domain boundary, consistent with previous studies.^1-4^


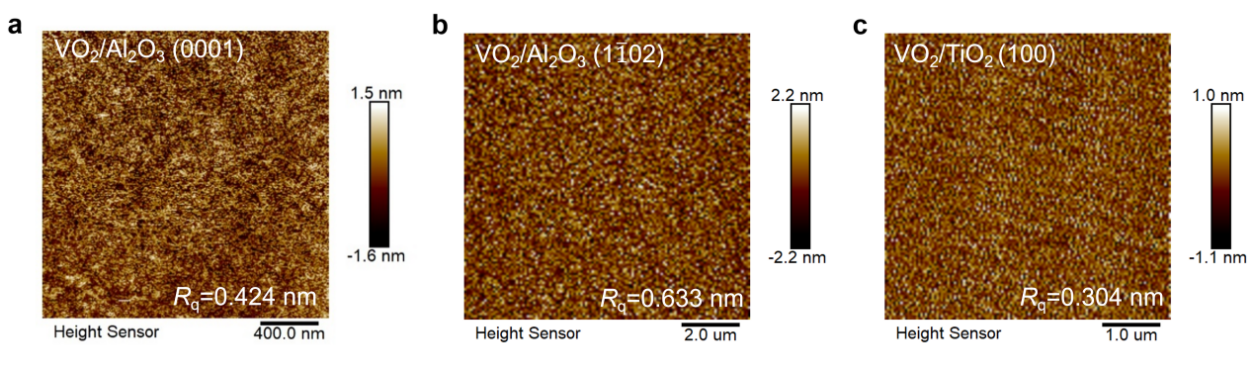


**Supplementary Figure 2.** Atomic force microscope (AFM) images for **a**, as-deposited VO_2_/Al_2_O_3_ (0001) heterostructure. **b**, VO_2_/Al_2_O_3_ (1$\bar{1}$02) heterostructure and **c**, VO_2_/TiO_2_ (100) heterostructure. The grown VO_2_ films on the (0001)-oriented Al_2_O_3_, (1$\bar{1}$02)-oriented Al_2_O_3_ and (100)-oriented TiO_2_ substrates exhibit the relatively smooth surface, achieving a root-mean-square roughness of less than 1 nm.


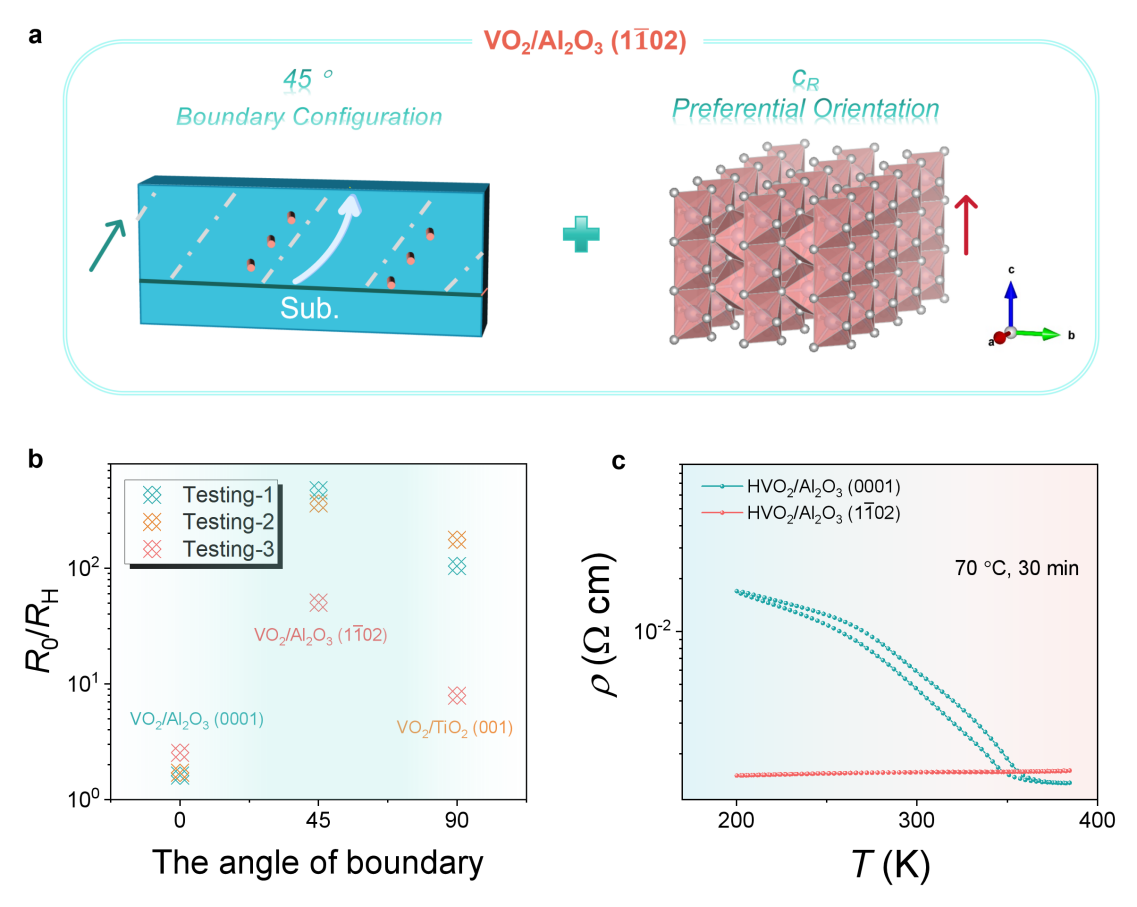


**Supplementary Figure 3. a**, Schematic of unobstructed freeway for hydrogen diffusion created by the 45º-tilted boundary configuration and *c*_R_-faceted preferential orientation. **b**, Hydrogen-triggered variation in the material resistivity (*R*_0_/*R*_H_) **c**, Temperature dependence of material resistivity (*ρ*-*T*) as measured for VO_2_ films through microstructure design as hydrogenated at 70 ºC for 30 min. It can be seen that the hydrogen-triggered electronic phase modulation achievable in the VO_2_/Al_2_O_3_ (1$\bar{1}$02) heterostructure, featured with an inclined domain boundary and *c*_R_-faceted preferential crystal orientation, is significantly expedited compared with the VO_2_/Al_2_O_3_ (0001) hybrid. With a relatively mild hydrogenation (e.g., 70 ºC, 30 min), the transport behavior for VO_2_ film deposited on the *r*-plane Al_2_O_3_ substrate resembles that of typical metals, which differs from the VO_2_/Al_2_O_3_ (0001) heterostructure, where the insulator-metal transition is still detectable. It is in particular worthy to note that the herein achievable electrical transport properties are consistent with the results shown in Figure 2b, demonstrating an excellent experimental reproducibility.


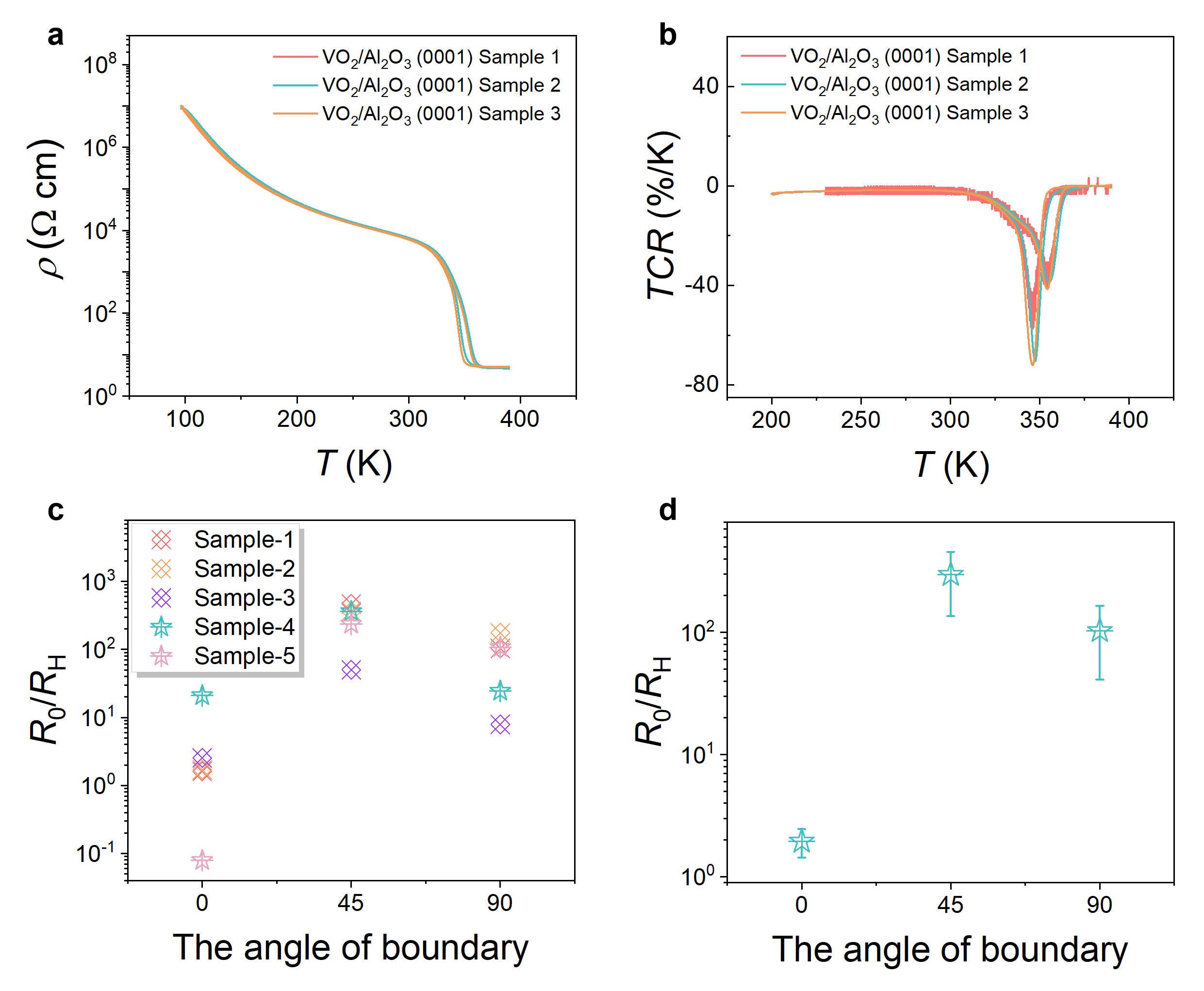


**Supplementary Figure 4. Reproducible thermally-driven and hydrogen-triggered electronic phase transitions in VO_2_ system: a**, *ρ*-*T* tendencies, **b**, *TCR*-*T* tendencies, and **c-d**, *R*_0_/*R*_H_ for different VO_2_ samples. The grown VO_2_ film deposition follows well-established protocols with stable growth conditions, where the discrepancy in the *T*_IMT_ for different VO_2_/Al_2_O_3_ (0001) samples is below 2 K. Although *R*_0_/*R*_H_ varies marginally across VO_2_ samples, independent experiments and statistical analyses affirm that hydrogen-triggered electronic phase transitions are accelerated in VO_2_/Al_2_O_3_ (1$\bar{1}$02) heterostructures with 45 º-tilted domain boundaries.


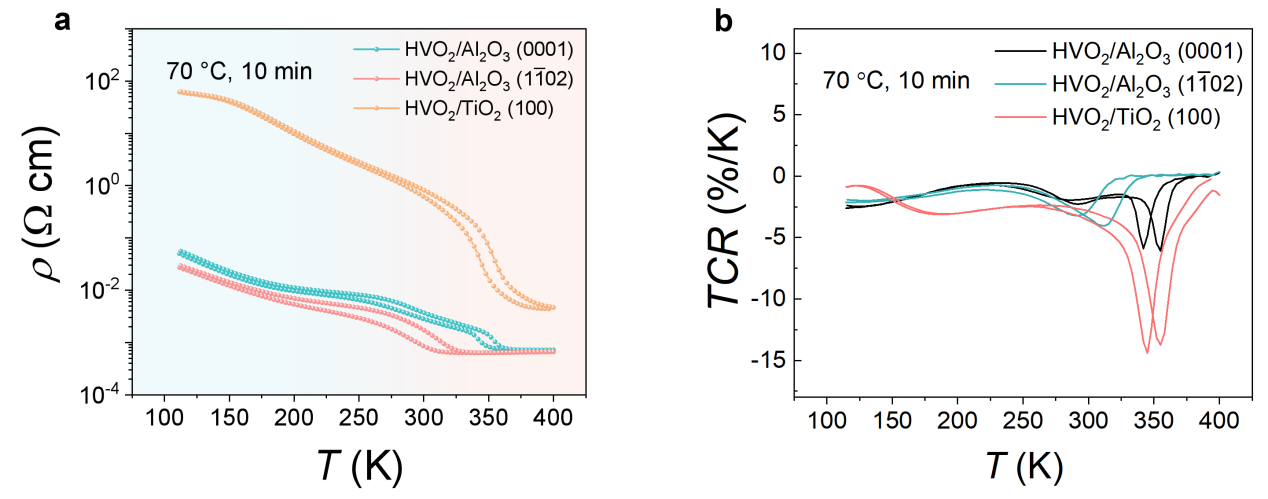


**Supplementary Figure 5. a**, Temperature dependence of material resistivity (*ρ*-*T*) as measured for VO_2_ with microstructure engineering as hydrogenated at 70 ºC for 10 min. **b**, Temperature coefficient of resistance (*TCR*) plotting as a function of temperature, as compared for VO_2_ with microstructure engineering. Upon the identical hydrogenation conditions, the transition temperature achievable in the VO_2_/Al_2_O_3_ (1$\bar{1}$02) heterostructure is significantly lower than the one for VO_2_ film grown on the Al_2_O_3_ (0001) substrate, as evidenced by the *TCR*-*T* tendency. This phenomenon indicates that the hydrogen-related electronic phase modulation for VO_2_/Al_2_O_3_ (1$\bar{1}$02) bilayer is faster than the widely-reported one grown on the *c*-plane Al_2_O_3_ substrate. Therefore, establishing an unobstructed highway for hydrogen diffusion can accelerate the electronic phase modulation of VO_2_ through hydrogenation.


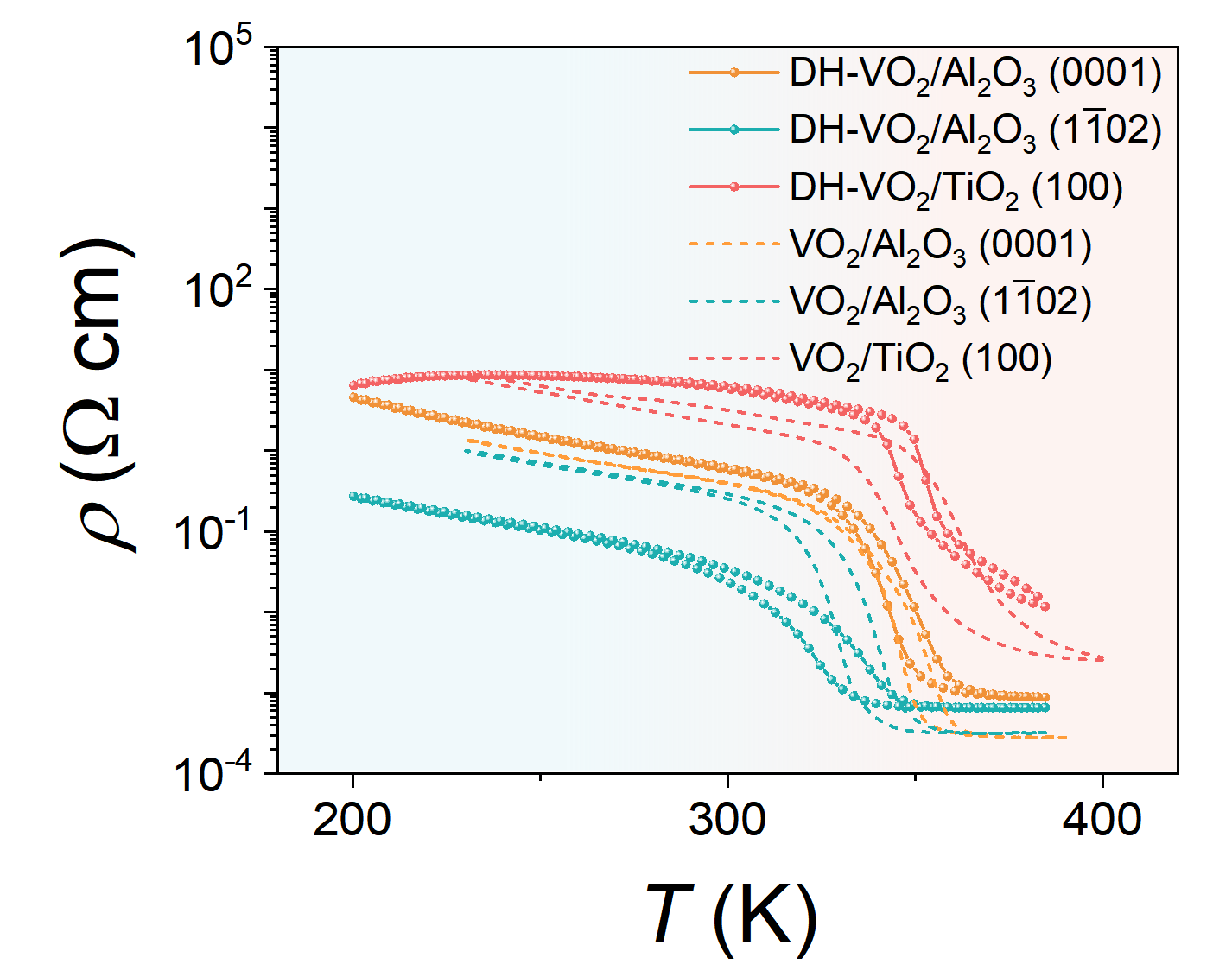


**Supplementary Figure 6.** Temperature dependence of material resistivity (*ρ*-*T*) as measured for dehydrogenated VO_2_ (denoted as DH-VO_2_) after annealing the hydrogenated VO_2_ at 70 ºC for 30 min. It is found that the expected electronic phase transitions of VO_2_ films grown on the Al_2_O_3_ (0001), Al_2_O_3_ (1$\bar{1}$02) and TiO_2_ (100) substrates are revived when annealing in an oxygen-rich atmosphere at 70 ºC for 30 min, unraveling the high reversibility in proton evolution.


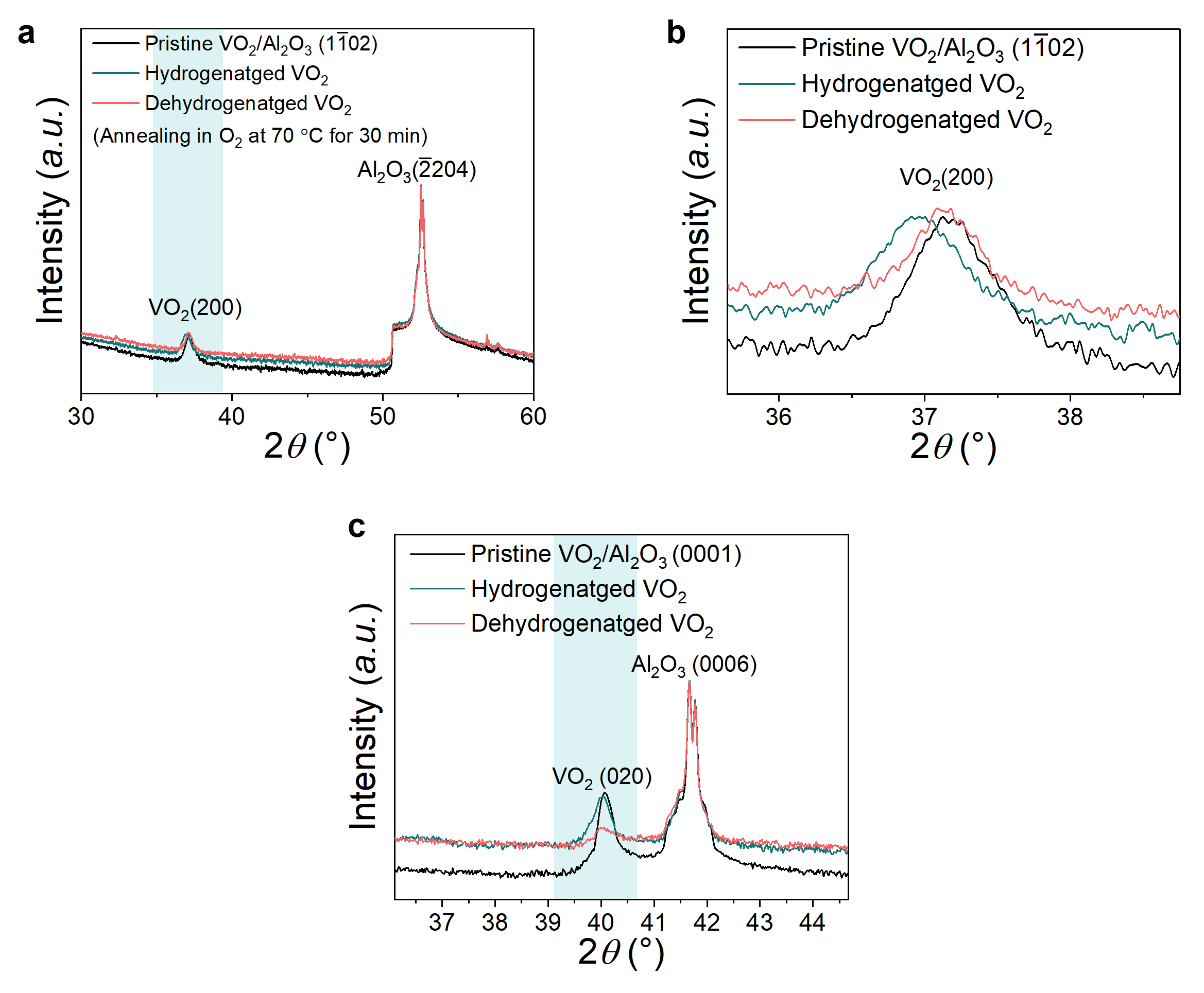


**Supplementary Figure 7. a**, X-ray diffraction (XRD) patterns compared for VO_2_/Al_2_O_3_ (1$\bar{1}$02) heterostructure upon hydrogenation and dehydrogenation. **b**, The zoom-in image of the XRD pattern for VO_2_/Al_2_O_3_ bilayer upon hydrogenation and dehydrogenation. **c**, XRD spectra compared for VO_2_/Al_2_O_3_ (0001) heterostructure upon hydrogenation and dehydrogenation. Performing the dehydrogenation process via annealing in an oxygen-rich atmosphere at 70 ºC for 30 min results in the recovery of both the hydrogenated phase and electrical transport property toward the initial state. The above results indicate reversible hydrogen-associated metallic electronic state of VO_2_.


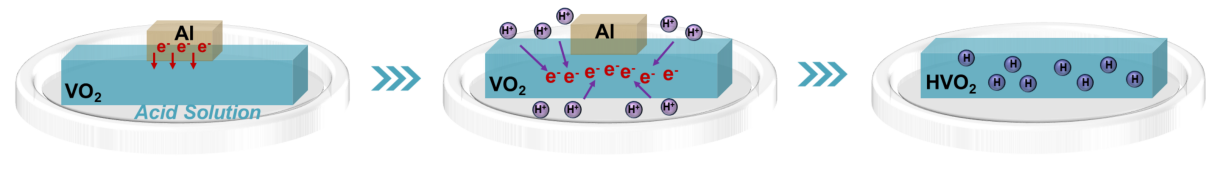


**Supplementary Figure 8.** Schematic of the metal-assisted acid solution strategy for achieving the hydrogenation of VO_2_. Here, the workfunction difference between the as-used metal (e.g., Al) and the VO_2_ renders the electron doping into the VO_2_ film, which simultaneously attracts the proton intercalation.^5-8^ Therefore, the hydrogenation of VO_2_ can be realized via electron-proton co-doping, free of involving the complex high-temperature process used in the hydrogen spillover strategy.


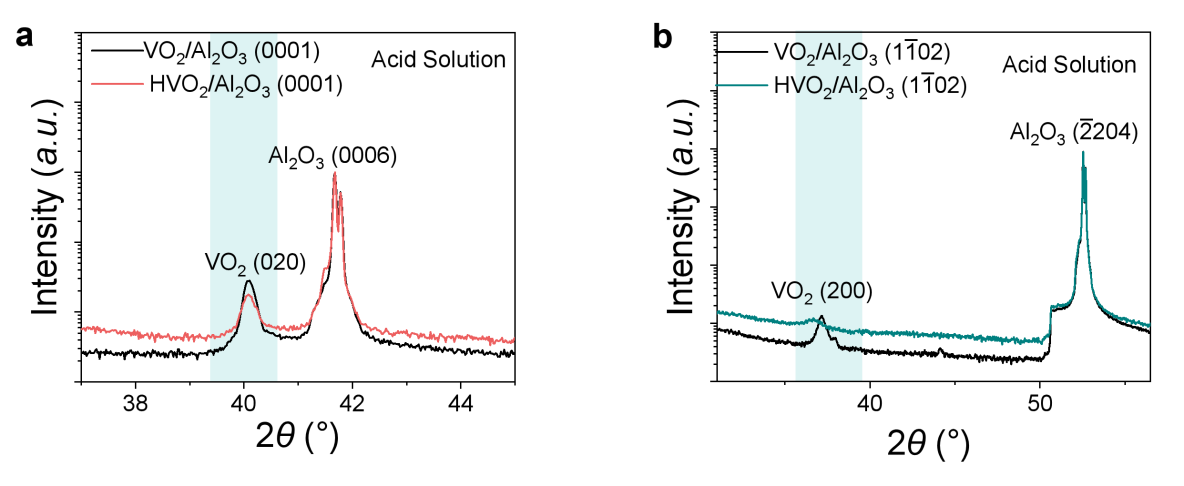


**Supplementary Figure 9.** X-ray diffraction (XRD) patterns compared for VO_2_ films deposited on the (0001) and (1$\bar{1}$02)-oriented Al_2_O_3_ substrates through acid-solution-triggered hydrogenation. It is found that the hydrogenation using the acid solution results in the lattice expansion of VO_2_ film, especially for the one deposited on the (1$\bar{1}$02)-oriented Al_2_O_3_ substrate.


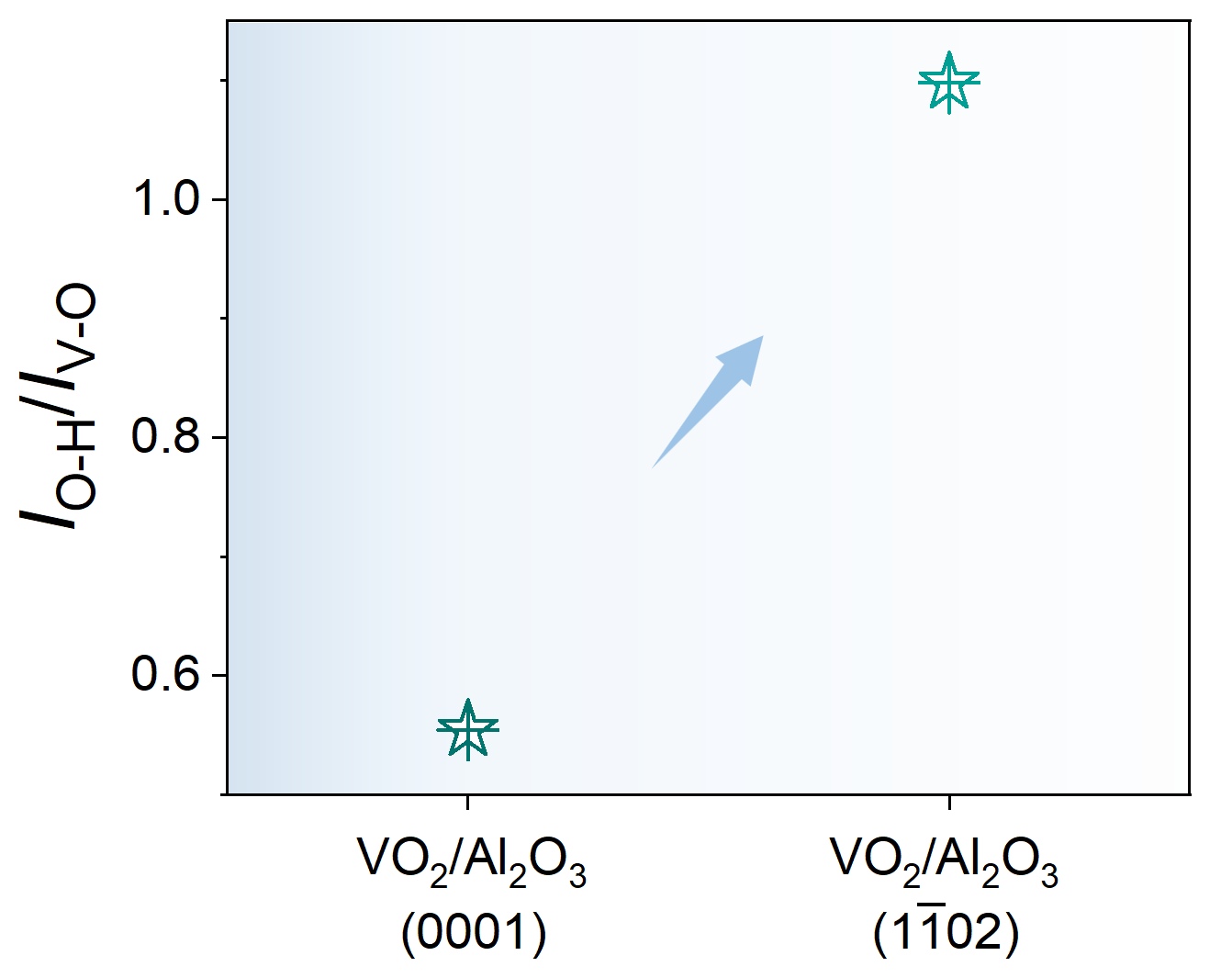


**Supplementary Figure 10.** The ratio of the peak intensity associated with the O-H and V-O interaction compared for hydrogenated VO_2_ grown on the *c*-plane and *r*-plane Al_2_O_3_ substrates, as characterized by using the X-ray photoelectron spectra (XPS). Upon hydrogenation, the elevation in the relative intensity of O-H interaction with respect to the V-O interaction further demonstrates that the incorporated hydrogens, occupying the interstitial sites, tend to bond with the lattice oxygen of VO_2_ to form such the O-H interactions.


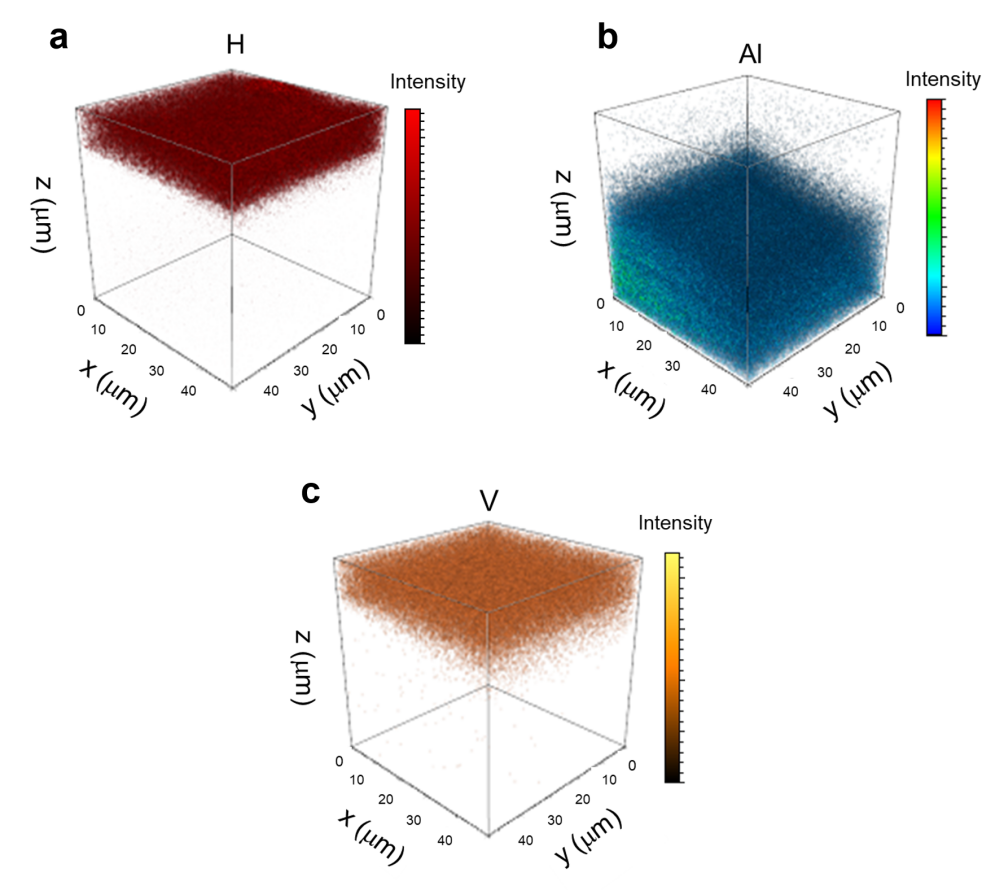


**Supplementary Figure 11.** Three-dimensional **a**, H **b**, Al and **c**, V element maps for hydrogenated VO_2_/Al_2_O_3_ (0001) bilayer as characterized by using the time-of-flight secondary ion mass spectrometry (ToF-SIMS) analysis. According to the ToF-SIMS result, the identical element distribution as observed for V and H elements demonstrates the effective incorporation of hydrogens into the lattice of VO_2_ film deposited on the *c*-plane Al_2_O_3_ substrate upon hydrogenating at 70 ºC for 30 min.


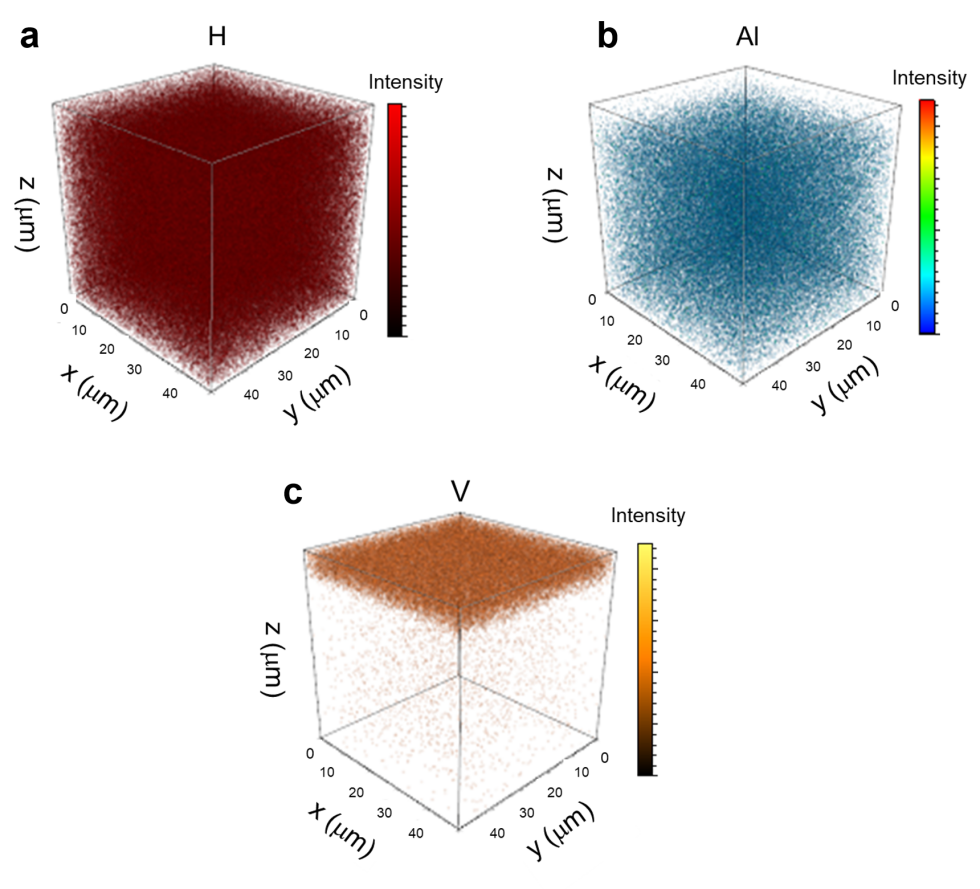


**Supplementary Figure 12.** Three-dimensional **a**, H **b**, Al and **c**, V element maps for hydrogenated VO_2_/TiO_2_ (001) bilayer as characterized by using the time-of-flight secondary ion mass spectrometry (ToF-SIMS) analysis. In contrast to the VO_2_ films deposited on the *c*-plane and *r*-plane Al_2_O_3_ substrate, no significant H signal discrepancy is observed for the grown VO_2_ film and the TiO_2_ (100) substrate, where the observed H signal approaches to the detection limit. This understanding regrading to the hydrogen distribution of VO_2_/TiO_2_ (100) heterostructure is more clearly demonstrated by Figure S12. This results demonstrable that the horizontally-aligned domain boundary as naturally formed in the VO_2_ film grown on the (100)-oriented TiO_2_ substrate could depress the proton evolution kinetics. Considering the herein employed hydrogenation condition (70ºC, 30 min) is fairly mild, no detectable H signal was observed in VO_2_/TiO_2_ (100) bilayer.


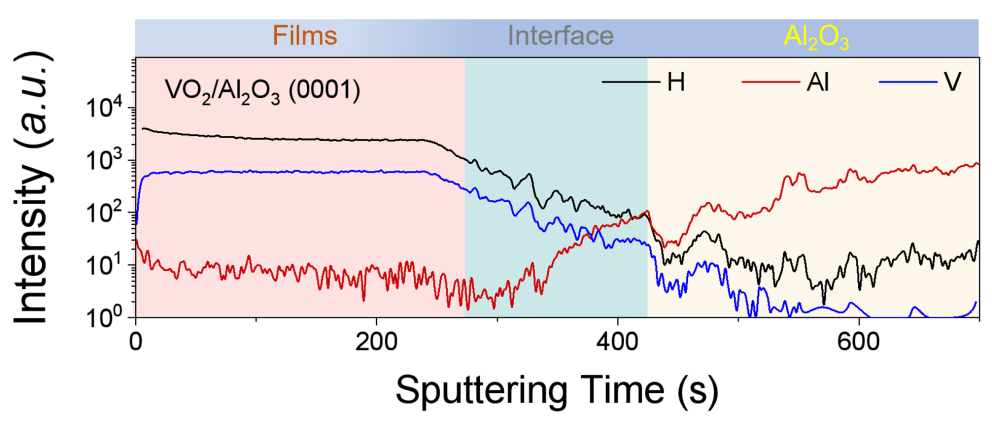


**Supplementary Figure 13.** Depth profile of the hydrogen, aluminum and vanadium elementary distribution within the lattice of VO_2_ film deposited on the Al_2_O_3_ (0001) substrate. In the depth profile of element distribution for hydrogenated VO_2_/Al_2_O_3_ (0001), the H signal in the VO_2_ film significantly exceeds that of Al_2_O_3_ substrate, demonstrating an effective hydrogen incorporation.


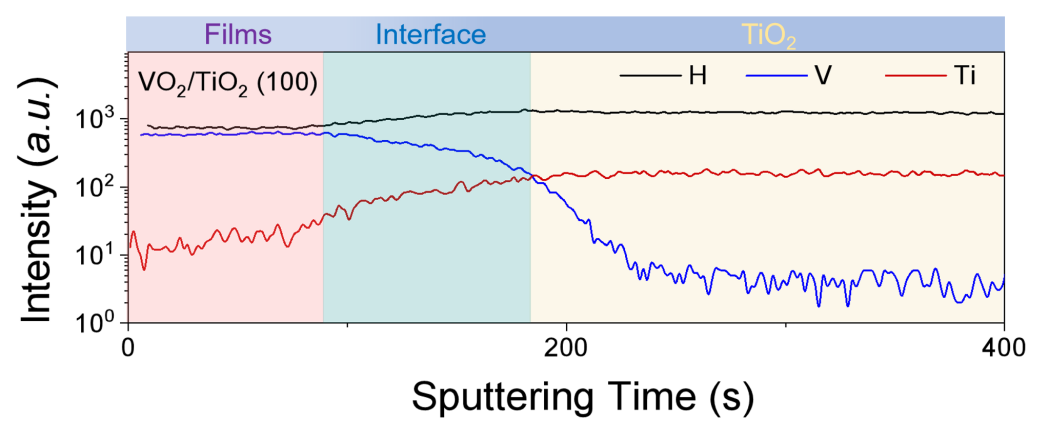


**Supplementary Figure 14.** Depth profile of the hydrogen, vanadium and titanium elementary distribution within the lattice of VO_2_ film deposited on the TiO_2_ (100) substrate. Considering similar H signal between the film and substrate regions, H signal is not detectable within the lattice of VO_2_, indicating a slow hydrogenation kinetics in the VO_2_/TiO_2_ (100) bilayer characterized by the horizontally-aligned domain boundary.


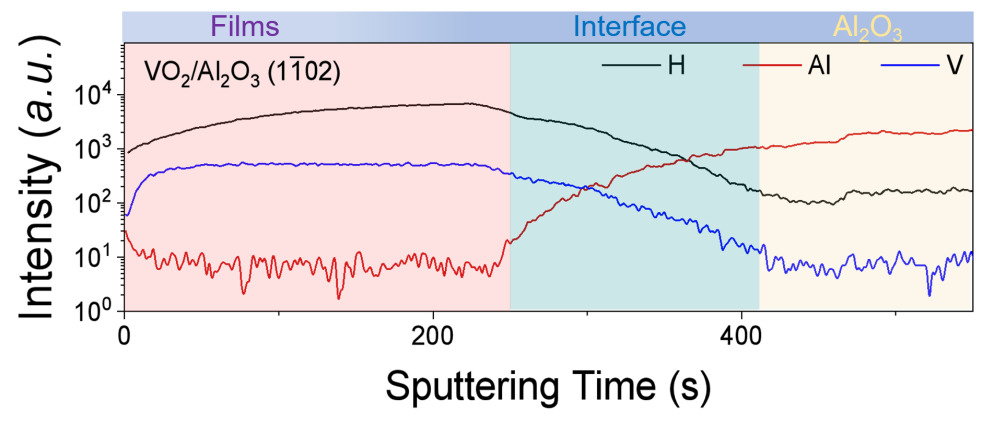


**Supplementary Figure 15.** Depth profile of the hydrogen, aluminum and vanadium elementary distribution within the lattice of VO_2_ film deposited on the Al_2_O_3_ (1$\bar{1}$02) substrate. The H signal is significantly higher compared with the widely reported VO_2_/Al_2_O_3_ (0001) and VO_2_/TiO_2_ (100) heterostructures, with an uphill hydrogen distribution being observed, where the hydrogen concentration progressively elevates with the diffusion length from the hydrogen source. The above uphill hydrogen distribution was previously observed in transitional metal oxides in previous reports, which was dominated by interfacial strain.^9, 10^ The unobstructed conduit for hydrogen diffusion, established by an inclined domain boundary and preferential *c*_R_-faceted crystal orientation, renders the anomalous hydrogen distribution that deviates from the Fick’s law. The above results further unveil an effective regulation in the hydrogenation kinetics and hydrogen-associated electronic phase modulations of VO_2_ through the artificial design of material microstructure.


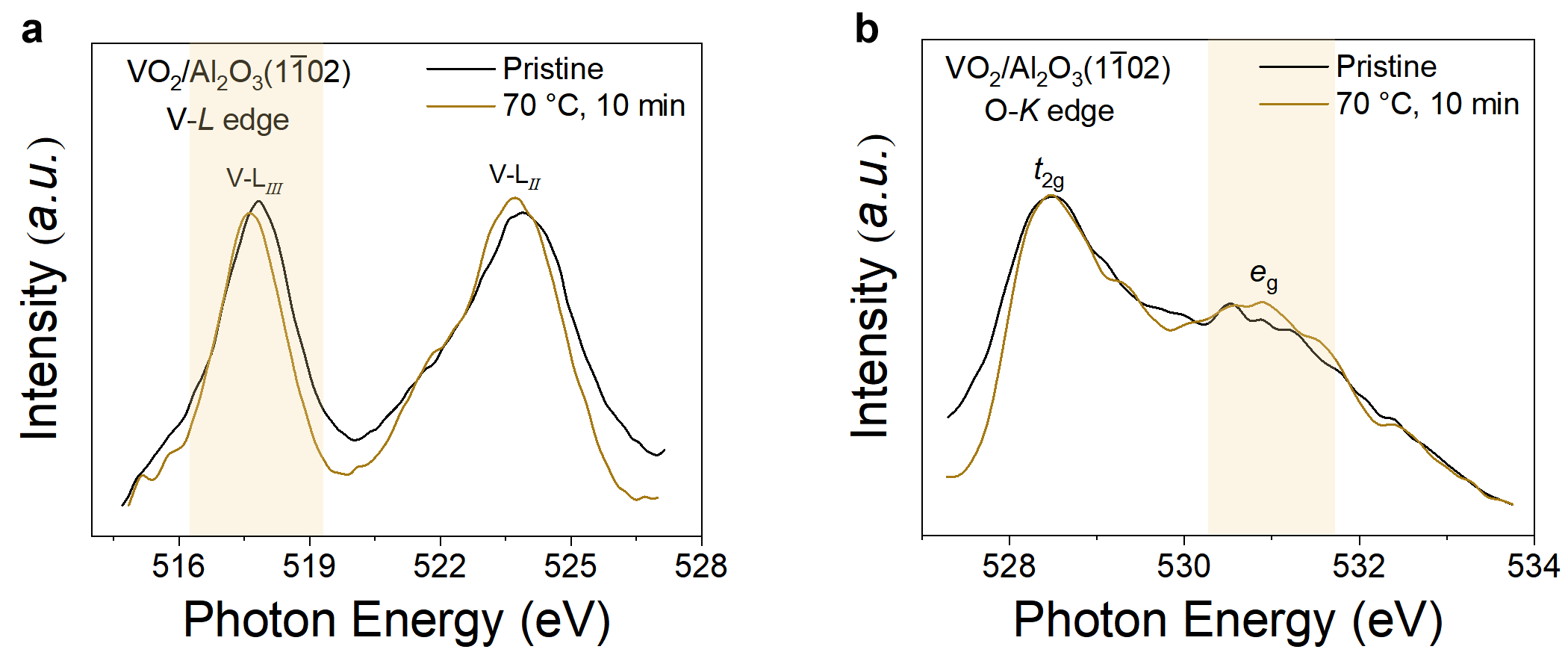


**Supplementary Figure 16.** Soft X-ray absorption spectroscopy (sXAS) for the **a**, V-*L* edge and **b**, O-*K* edge of VO_2_/Al_2_O_3_ (1$\bar{1}$02) heterostructure through hydrogenation at 70 ºC for 10 min. It can be seen that the valence state of vanadium of VO_2_ reduces through hydrogenation, while the hydrogen-associated electron doping in the *t*_2g_ band is unveiled, according to previous works.^11-15^


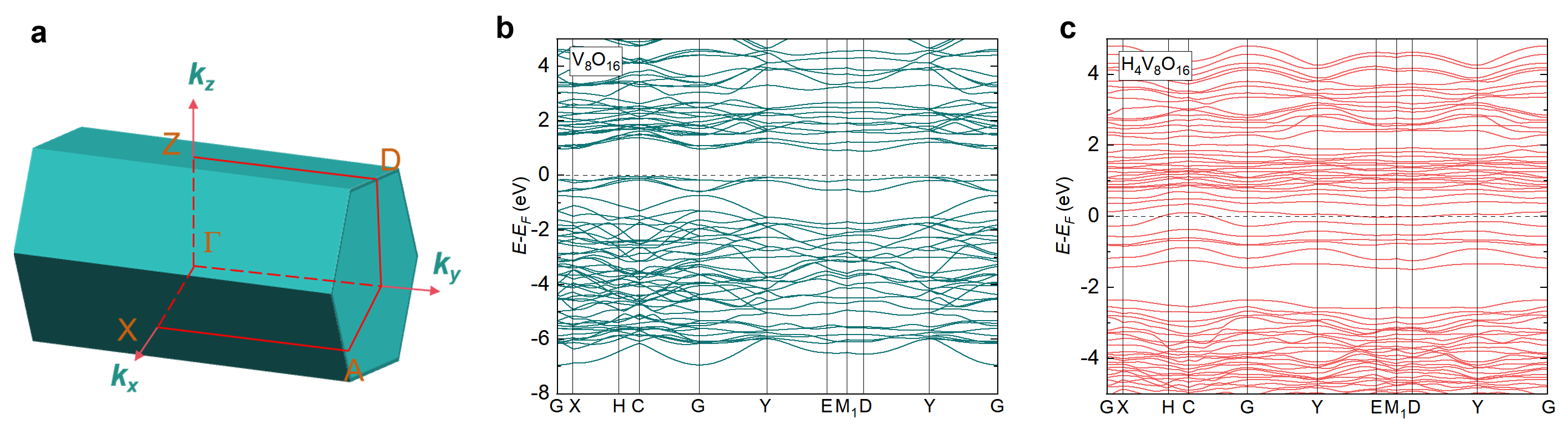


**Supplementary Figure 17.** **a**, Schematic of the first Brillouin zone. **b-c**, Calculated band structure for **b**, pristine VO_2_, and **c**, hydrogenated VO_2_ (H_4_V_8_O_16_). Utilizing the DFT calculations, the hydrogen-associated electronic phase modulation from correlated electronic ground state to electron-itinerant state is clearly demonstrated through hydrogenation.


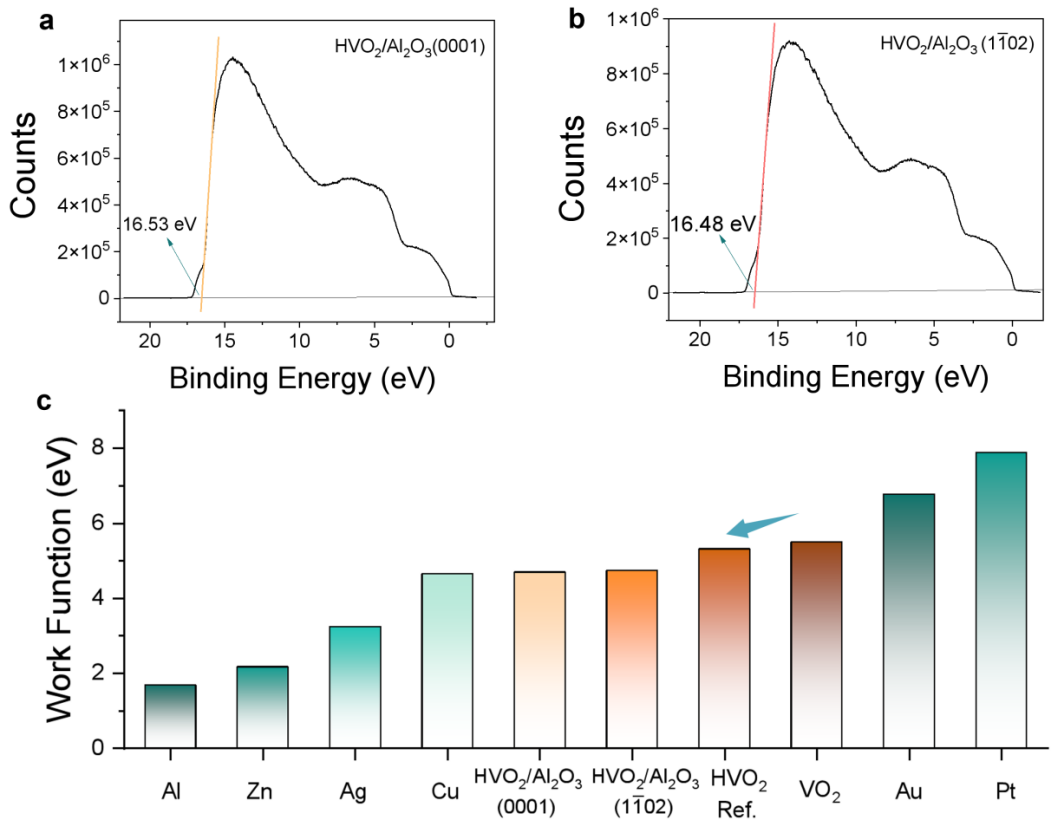


**Supplementary Figure 18.** **a-b**, Ultraviolet photoelectron spectroscopy (UPS) spectra compared for the **a**, VO_2_/Al_2_O_3_ (0001) and **b**, VO_2_/Al_2_O_3_ (1$\bar{1}$02) bilayer through hydrogenation at 70 ºC for 30 min. **c**, Work function of the hydrogenated VO_2_ deposited on the *c*-plane and *r*-plane Al_2_O_3_ substrates, compared with the previous report. Utilizing the UPS analysis, the workfunction of hydrogenated VO_2_ grown on the Al_2_O_3_ (1$\bar{1}$02) and Al_2_O_3_ (0001) substrates are estimated to be 4.74 eV and 4.69 eV, respectively. Hydrogenation or protonation effectively reduces the workfunction of VO_2_ from ~5.12-5.85 eV to ~4.69-4.74 eV, owing to the band filling in the low-energy *t*_2g_ orbital, analogous to the previous report.^5^

**Table S1** Comparing the lattice expansion for VO_2_ films through hydrogenation.

| Sample | Peak position of pristine VO_2_ | Peak position of hydrogenated VO_2_ | Variation |
| --- | --- | --- | --- |
| VO_2_/Al_2_O_3_ (1$\bar{1}$02) | 37.2 | 36.95 | 0.25 |
| VO_2_/Al_2_O_3_ (0001) | 40.1 | 40.06 | 0.04 |
| VO_2_/TiO_2_ (100) | 40.31 | 40.24 | 0.09 |

**Table S2** Comparing the microstructure of VO_2_ films deposited on different substrates.

| Sample | Domain boundary configuration | Preferential orientation |
| --- | --- | --- |
| VO_2_/Al_2_O_3_ (1$\bar{1}$02) | 45 º tilted | *c*_R_-faceted |
| VO_2_/Al_2_O_3_ (0001) | 90 º tilted | *b*_M_-faceted |
| VO_2_/TiO_2_ (100) | 0 º tilted | *a*_R_-faceted |

**Table S3** The resistive switching for different VO_2_ samples through hydrogenation.

| Sample | *R*_0_/*R*_H_ Sample-1 | *R*_0_/*R*_H_ Sample-2 | *R*_0_/*R*_H_ Sample-3 | *R*_0_/*R*_H_ Sample-4 | *R*_0_/*R*_H_ Sample-5 |
| --- | --- | --- | --- | --- | --- |
| VO_2_/Al_2_O_3_ (1$\bar{1}$02) | 471.01449 | 362.84939 | 50.312 | 362.01117 | 236.54345 |
| VO_2_/Al_2_O_3_ (0001) | 104.16667 | 176.06392 | 7.913 | 24.46809 | 107.67612 |
| VO_2_/TiO_2_ (100) | 1.60714 | 1.70146 | 2.535 | 21 | 0.07864 |

**Table S4** The transition temperature (*T*_MIT_) for different VO_2_/Al_2_O_3_ (0001) samples.

| VO_2_/Al_2_O_3_ (0001)  Sample | *T*_MIT_  Heating (K) | *T*_MIT_  Cooling (K) | *T*_MIT_ (K) |
| --- | --- | --- | --- |
| #1 | 354.264 | 345.657 | 349.9605 |
| #2 | 354.948 | 347.310 | 351.1290 |
| #3 | 354.264 | 345.942 | 350.1030 |

**Table S5** The incorporated hydrogen concentration in VO_2_ films through hydrogenation.

| Sample | Average hydrogen concentration in film region | Average hydrogen concentration in substrate region | Hydrogen concentration in VO_2_ film |
| --- | --- | --- | --- |
| VO_2_/Al_2_O_3_ (1$\bar{1}$02) | 4653.21 | 103.73 | 4549.48 |
| VO_2_/Al_2_O_3_ (0001) | 2604.16 | 37.72 | 2566.44 |
| VO_2_/TiO_2_ (100) | 1266.59 | 1150.87 | 115.72 |

**Supplementary Note 1 Manipulating the IMT of VO_2_ through critical temperature and hydrogenation.**

The IMT properties of VO_2_ can be triggered and adjusted through critical temperature and hydrogen doping:

1. Thermally-driven IMT: Conventionally, insulating VO_2_ with a monoclinic phase (M1) can transit to the rutile metallic phase of VO_2_ via the splitting of *d*_//_ orbital and/or the V-V dimerization across the critical temperature (e.g., *T*_IMT_).
2. Hydrogen-driven IMT: Hydrogen doping introduces an additional pathway to trigger electronic phase transition of VO_2_ at room temperature, transitioning the insulating ground state of VO_2_ based on *t*_2g_^1^*e*_g_^0^ state towards metallic state based on *t*_2g_^1+Δ^*e*_g_^0^ configuration. Such the proton evolution initially depresses the IMT of VO_2_, followed by the collective metallization. Accompanied by the soft-chemistry hydrogen-spillover H_2_ (g)→ H^+^+e^-^ reaction, each hydrogen can donate one electron into VO_2_, directly altering d-orbital filling and configuration. Hydrogen-mediated Mott phase modulations in VO_2_ system are attributed to the band filling in empty t_2g_ orbital that reconfigures electronic band structure, which are recognized as pure Mottronic phase transition. In particular, the ionic interactions between the intercalated hydrogens as interstitial atoms and extended planer defect provides a feasible pathway to adjust the proton evolution process that accelerates or depresses hydrogenation kinetics. Our findings highlight a powerful tuning knob for tailoring the ionic evolution in VO_2_ system through artificial microstructure design.

**Supplementary References**

1. Park J, Yoon H, Sim H, Choi SY, Son J. Accelerated Hydrogen Diffusion and Surface Exchange by Domain Boundaries in Epitaxial VO_2_ Thin Films. *ACS Nano* **14**, 2533-2541 (2020).

2. Zhao Y*, et al.* Structural, electrical, and terahertz transmission properties of VO_2_ thin films grown on c-, r-, and m-plane sapphire substrates. *J Appl Phys* **111**, 053533 (2012).

3. Qiao L, Xiao HY, Weber WJ, Biegalski MD. Coexistence of epitaxial lattice rotation and twinning tilt induced by surface symmetry mismatch. *Appl Phys Lett* **104**, 221602 (2014).

4. Yang T-H, Aggarwal R, Gupta A, Zhou H, Narayan RJ, Narayan J. Semiconductor-metal transition characteristics of VO_2_ thin films grown on c- and r-sapphire substrates. *J Appl Phys* **107**, 053514 (2010).

5. Chen YL*, et al.* Non-catalytic hydrogenation of VO_2_ in acid solution. *Nat Commun* **9**, 818 (2018).

6. Li B*, et al.* Electron-Proton Co-doping-Induced Metal-Insulator Transition in VO_2_ Film via Surface Self-Assembled l-Ascorbic Acid Molecules. *AngewChem Int Edit* **58**, 13711-13716 (2019).

7. Ren H*, et al.* Controllable Strongly Electron-Correlated Properties of NdNiO_3_ Induced by Large-Area Protonation with Metal-Acid Treatment. *ACS Appl Electron Mater* **4**, 3495-3502 (2022).

8. Xie LY*, et al.* Tunable Hydrogen Doping of Metal Oxide Semiconductors with Acid-Metal Treatment at Ambient Conditions. *J Am Chem Soc* **142**, 4136-4140 (2020).

9. Fayaz MU, Wang Q, Xu M, Chen D, Pan F, Song C. Compressive Strain-Induced Uphill Hydrogen Distribution in Strontium Ferrite Films. *ACS Appl Mater Interfaces* **17**, 21371–21379 (2025).

10. Wang Q*, et al.* Strain-Induced Uphill Hydrogen Distribution in Perovskite Oxide Films. *ACS Appl Mater Interfaces* **16**, 3726-3734 (2024).

11. Zhou X*, et al.* Revealing the Role of Hydrogen in Electron-Doping Mottronics for Strongly Correlated Vanadium Dioxide. *J Phys Chem Lett* **13**, 8078-8085 (2022).

12. Zhou X*, et al.* Revealing the role of high-valence elementary substitution in the hydrogen-induced Mottronic transitions of vanadium dioxide. *Appl Phys Lett* **124**, 082103 (2024).

13. Deng X*, et al.* Spatial evolution of the proton-coupled Mott transition in correlated oxides for neuromorphic computing. *Sci Adv* **10**, eadk9928 (2024).

14. Chen S*, et al.* Sequential insulator-metal-insulator phase transitions of VO_2_ triggered by hydrogen doping. *Phys Rev B* **96**, 125130 (2017).

15. Deng S*, et al.* Selective area doping for Mott neuromorphic electronics. *Sci Adv* **9**, eade4838 (2023).
